# Lipidated Calcitonin Gene-Related Peptide (CGRP) Peptide Antagonists Retain CGRP Receptor Activity and Attenuate CGRP Action *In Vivo*

**DOI:** 10.3389/fphar.2022.832589

**Published:** 2022-03-07

**Authors:** Aqfan Jamaluddin, Chia-Lin Chuang, Elyse T. Williams, Andrew Siow, Sung Hyun Yang, Paul W. R. Harris, Jakeb S. S. M. Petersen, Rebekah L. Bower, Shanan Chand, Margaret A. Brimble, Christopher S. Walker, Debbie L. Hay, Kerry M. Loomes

**Affiliations:** ^1^ School of Biological Sciences, University of Auckland, Auckland, New Zealand; ^2^ School of Chemical Sciences, University of Auckland, Auckland, New Zealand; ^3^ Department of Pharmacology and Toxicology, University of Otago, Dunedin, New Zealand

**Keywords:** CGRP, lipidation, AMY_1_, peptide, migraine, vasodilation, GPCR

## Abstract

Signaling through calcitonin gene-related peptide (CGRP) receptors is associated with pain, migraine, and energy expenditure. Small molecule and monoclonal antibody CGRP receptor antagonists that block endogenous CGRP action are in clinical use as anti-migraine therapies. By comparison, the potential utility of peptide antagonists has received less attention due to suboptimal pharmacokinetic properties. Lipidation is an established strategy to increase peptide half-life *in vivo*. This study aimed to explore the feasibility of developing lipidated CGRP peptide antagonists that retain receptor antagonist activity *in vitro* and attenuate endogenous CGRP action *in vivo*. CGRP peptide analogues based on the archetypal CGRP receptor antagonist, CGRP_8-37_, were palmitoylated at the N-terminus, position 24, and near the C-terminus at position 35. The antagonist activities of the lipidated peptide analogues were tested *in vitro* using transfected Cos-7 cells expressing either the human or mouse CGRP receptor, amylin subtype 1 (AMY_1_) receptor, adrenomedullin (AM) receptors, or calcitonin receptor. Antagonist activities were also evaluated in SK-N-MC cells that endogenously express the human CGRP receptor. Lipidated peptides were then tested for their ability to antagonize endogenous CGRP action *in vivo* using a capsaicin-induced dermal vasodilation (CIDV) model in C57/BL6J mice. All lipidated peptides except for the C-terminally modified analogue retained potent antagonist activity compared to CGRP_8-37_ towards the CGRP receptor. The lipidated peptides also retained, and sometimes gained, antagonist activities at AMY_1_, AM_1_ and AM_2_ receptors. Several lipidated peptides produced robust inhibition of CIDV in mice. This study demonstrates that selected lipidated peptide antagonists based on αCGRP_8-37_ retain potent antagonist activity at the CGRP receptor and are capable of inhibition of endogenous CGRP action *in vivo*. These findings suggest that lipidation can be applied to peptide antagonists, such as αCGRP_8-37_ and are a potential strategy for antagonizing CGRP action.

## Introduction

Calcitonin gene related peptide (CGRP) is a 37 amino acid neuropeptide peptide belonging to the calcitonin family of peptides comprising adrenomedullin (AM) 1 and 2, amylin and calcitonin (Hay et al., 2018). CGRP exists as αCGRP and βCGRP isoforms, differing by three amino acids in humans and two amino acids in rodents. Both peptides are expressed in the central and peripheral nervous systems, with βCGRP having a particular prominence in the enteric nervous system (Mulderry et al., 1988; Sternini, 1992).

The receptors that mediate the actions of the calcitonin peptide family are heterodimeric and comprise either the calcitonin receptor-like receptor (CLR) or calcitonin receptor (CTR) in complex with one of the three receptor activity-modifying proteins (RAMPs). The CGRP receptor (CLR:RAMP1) is considered the canonical receptor, and signals primarily through the adenylyl cyclase pathway (Bailey and Hay, 2006). Other combinations produce additional receptors such as AM_1_ (CLR/RAMP2), AM_2_ (CLR/RAMP3), AMY_1_ (CTR/RAMP1), AMY_2_ (CTR/RAMP2) and AMY_3_ (CTR/RAMP3) receptors (Hay et al., 2018). CGRP also binds and activates the AMY_1_ receptor with equal potency to the amylin peptide. By comparison, CGRP is significantly less potent at the AM_1_ and AM_2_ receptors and has very weak activity at the CTR (Hay et al., 2018; Garelja et al., 2020).

Of the two CGRP isoforms, attention has mostly focused historically on αCGRP and its role in diverse physiological processes such as vasodilation (Greenberg et al., 1987; Gray and Marshall, 1992), inflammation (Brain and Williams, 1985; Basbaum et al., 2009), cardiovascular conditioning (Liu et al., 2011; Mishima et al., 2011; Smillie et al., 2014), energy homeostasis (Walker et al., 2010; Bartelt et al., 2017; Liu et al., 2017) and sensory nerve functions (Walker et al., 2015; Eftekhari et al., 2016). αCGRP is a potent vasodilator, producing skin reddening *in vivo* evoked by intradermal administration of αCGRP (Brain and Williams, 1985). This vasodilatory effect is mediated through cAMP-dependent pathway signaling (Brain and Grant, 2004). Of particular clinical significance is CGRP’s etiological role in migraine (Edvinsson, 2018). Systemic administration of αCGRP can provoke migraine-like attacks in migraineurs (Lassen et al., 2002; Hansen et al., 2010; Asghar et al., 2011; Guo et al., 2016).

A range of CGRP antagonist therapeutics comprising monoclonal antibodies, and small molecules are now approved clinically for the treatment of migraine. Currently, there are four approved monoclonal antibodies that block CGRP activity as preventative treatments for migraine. The first approved human monoclonal antibody, erenumab (AMG-334), targets the canonical CGRP receptor (Shi et al., 2016) with clinical efficacy (Sun et al., 2016; Goadsby et al., 2017). This was soon followed by approval of fremanezumab (LBR-101/TEV-48125) targeting CGRP itself (Bigal et al., 2015; Dodick et al., 2018). Two other monoclonal antibodies targeting the CGRP peptide, galcanezumab (LY2951742) and eptinezumab (ALD403) are also approved for preventative treatment of migraine. These antibody therapies are now complemented with the small molecule CGRP receptor antagonists, rimegepant (Croop et al., 2019), ubrogepant (Ailani et al., 2020) and atogepant (Schwedt et al., 2021) as approved acute treatments.

In addition to the development of antibodies and small molecules as CGRP antagonists, there may be opportunities to develop a new class of therapeutics with peptide-based antagonism. CGRP is modified post-translationally with a C-terminal amide and a cysteine-bridge between position 2 and 7 to confer a cyclic N-terminus. Truncation of the first seven amino acid residues of αCGRP yields αCGRP_8-37_, the archetypal competitive peptide antagonist to the CGRP receptor (Chiba et al., 1989). Shorter peptide fragments have also been reported with αCGRP_27-37_ being the shortest that retains antagonist activity at the CGRP receptor (Yan et al., 2011).

Peptide therapeutics that have similar properties to endogenous peptides are a particularly attractive drug class due to their safety profile (Muttenthaler et al., 2021). Nevertheless, there are intrinsic translational difficulties with CGRP peptide antagonists due to the short plasma half-life of CGRP and metabolic instability of αCGRP_8-37_ (Kraenzlin et al., 1985; Miranda et al., 2008; Struthers et al., 1986; Srinivasan et al., 2022). In attempts to overcome these deficiencies, analogues based on αCGRP_8-37_ and αCGRP_27-37_ have been developed ranging from N-terminal modification (Taylor et al., 2006) through to systematic amino acid substitutions, utilization of unnatural amino acids, peptide cyclization (Srinivasan et al., 2022), chimeric CGRP species and PEGylation (Struthers et al., 1986; Miranda et al., 2008). However, despite some reported improvements in pharmacokinetic profile compared to αCGRP_8-37_ (Miranda et al., 2013; Srinivasan et al., 2022), no CGRP peptide-based antagonist therapeutics have progressed to human clinical trials.

Peptide lipidation offers another attractive strategy of extending peptide half-life and has been used successfully in therapeutic development (Davies et al., 2015). In the present study we investigated whether it is possible to develop lipidated analogues based on CGRP_8-37_ that retain antagonist activities at the CGRP receptor *in vitro* and also attenuate CGRP action *in vivo*. We report the characterization of several cysteine-substituted CGRP_8-37_ analogues modified at various positions with a palmitoyl fatty acid sidechain. Our findings show that it is possible to lipidate αCGRP_8-37_ and retain antagonist activity at CGRP and AMY_1_ receptors but also increase potency in some cases. We also demonstrate successful antagonism of CGRP action *in vivo* by lipidated αCGRP_8-37_ analogues using a dermal vasodilatory model, suggesting lipidation of peptide antagonists could be a potential strategy to antagonize CGRP action.

## Materials and Methods

### Commercial Peptides and Antagonists

The following peptides were purchased commercially: human (h) and mouse (m) αCGRP, hAM and hAM_22-52_ (American Peptide, Sunnyvale, CA, United States, or Bachem, Bubendorf, Switzerland); calcitonin and salmon (s) calcitonin_8-32_ (sCT_8-32_) (American Peptide); αCGRP_8-37_ (American Peptide). Commercial αCGRP_8-37_ was used as a control to validate in-house synthesized αCGRP_8-37_. Olcegepant was purchased from AbaChemScene (NJ, United States).

### In House-Peptide Synthesis

hαCGRP_8-37_ together with cysteine-substituted analogues were synthesized with an amidated C-terminus using Fmoc solid-phase peptide synthesis (SPPS). Lipidation of peptides was synthesized by Solid-Phase Cysteine Lipidation of Peptides or Amino acids (SP-CLipPA) (Williams et al., 2018) or through the building block method (Lu et al., 2020). hβCGRP_8-37_, hαCGRP_8-37_ R11C-palmitate (R11C-palmitate), and hβCGRP_8-37_ V8C-palmitate (βV8C-palmitate) synthesis information can also be found in the Supplementary Data. All peptides were purified by RP-HPLC to ≥90% purity before lyophilization. For *in vitro* studies, non-lipidated hCGRP_8-37_ analogues and small molecule compounds were reconstituted as stock solutions in water or dimethyl sulfoxide (DMSO). Lipidated hCGRP_8-37_ analogues were reconstituted as stock solutions in 100% DMSO.

### Cell Culture and Transfection

Cos-7 cells and SK-N-MC cells were cultured in Dulbecco’s Modified Eagle Medium supplemented with 7.5% heat-inactivated fetal bovine serum. Cells were grown in a humidified incubator at 37°C and 5% CO_2_ and seeded into 96-well plates at a density of 20,000 cells/well. The following day, Cos-7 cells were transiently transfected using Polyethylenimine (PEI) using a 1:1 ratio of receptor:RAMP DNA. HA-tagged hCLR (Hay et al., 2003) or HA-tagged hCTR (CT_(a)_ splice variant, Udawela et al., 2006) in combination with either myc-tagged hRAMP1 (Qi et al., 2008), FLAG-tagged hRAMP2 (Qi et al., 2013), or untagged hRAMP3 (Hay et al., 2003) were transfected to express the desired calcitonin-family receptor. Untagged mCT_(a)_, mCLR, mRAMP1, mRAMP2 and mRAMP3 were purchased from Origene and transfected in an identical method to the human receptors into Cos-7 cells (Garelja et al., 2021).

### cAMP Assay Measurement

Transfected Cos-7 cells were incubated with agonist in the presence or absence of antagonist at 37°C for 15 min. cAMP production was terminated by aspirating all the media in the wells, followed by the addition of 50 μl of ice-cold ethanol. Cell lysates were then prepared for cAMP measurements using LANCE cAMP assay kit (Perkin Elmer, Waltham, MA, United States), as previously described (Woolley et al., 2017).

In one modification of the experimental design, cAMP content was investigated under conditions where the antagonist was added but then removed prior to agonist stimulation by αCGRP. Here, media in the 96-well plate was replaced with 50 μl serum-free DMEM containing 1 mM IBMX and 0.1% w/v bovine serum albumin (BSA) for 30 min at 37°C. After this period, 25 μl of the selected antagonist or media was added and pre-incubated with the transfected Cos-7 cells for 15 min at 37°C. The pre-incubated mixture was then removed by aspirating media from the selected wells, which were then washed once with 50 μl of phosphate-buffered saline and replaced with 75 μl of new serum-free DMEM containing 1 mM IBMX and 0.1% w/v BSA. Finally, 25 μl of the αCGRP agonist was added to each well, to a maximum volume of 100 μl and incubated for a further 15 min at 37°C. The agonist profiles were then compared to the condition-matched antagonist profiles.

### Animal Welfare and Ethical Statement

All studies involving animals were approved by the University of Auckland Animal Ethics Committee and conducted in accordance with the New Zealand animal welfare act (1999). Prior to the experiments, mice were housed in environmentally enriched cages under climatically controlled conditions and kept in a 12-h day/night cycle. Mice had ad libitum access to standard chow (Teklad TB 2018; Harlan, Madison, WI, United States) and water.

### Capsaicin-Induced Dermal Vasodilation - Laser Doppler Imaging Overview

**FIGURE 1:**
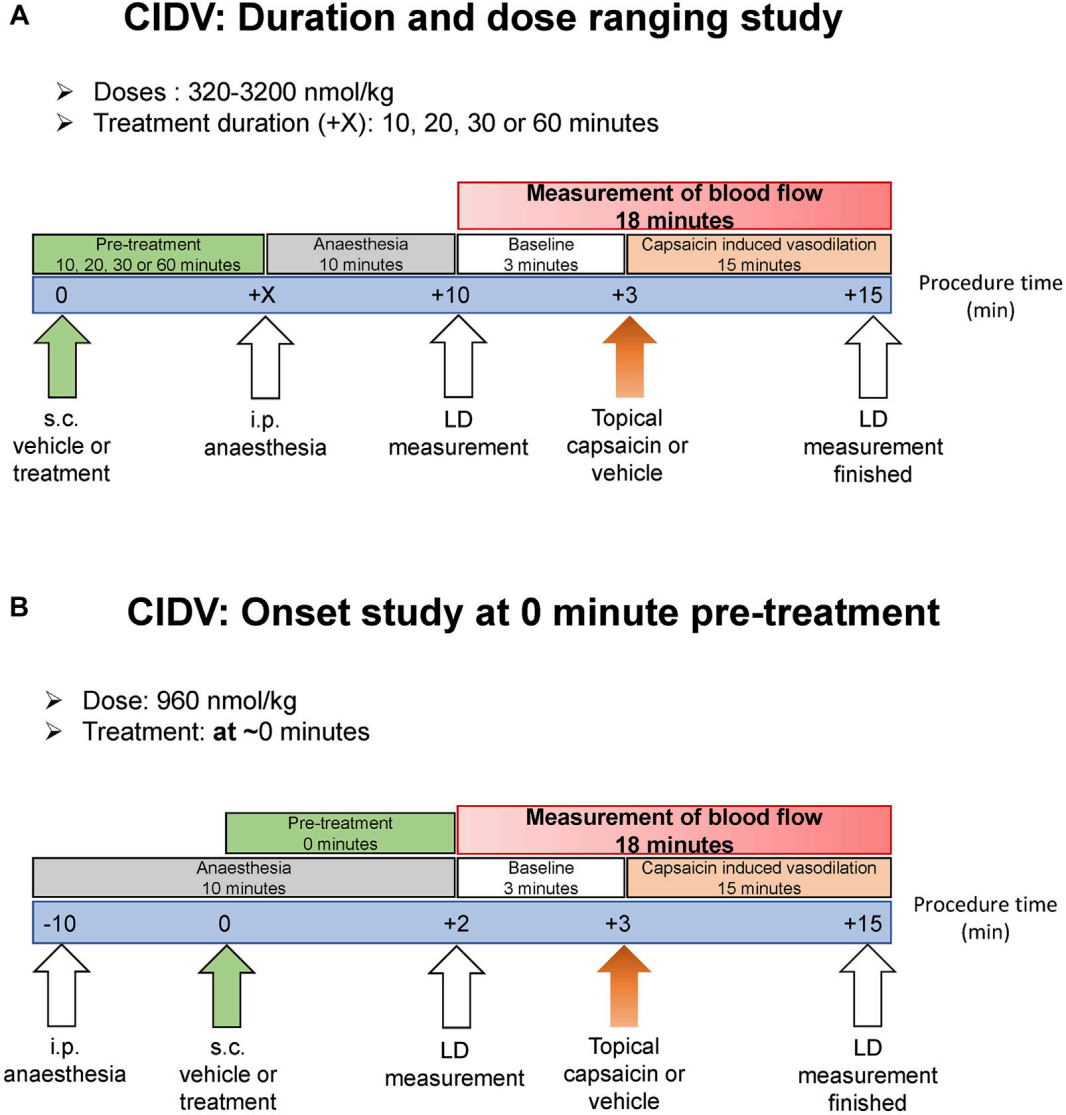
Schematic showing the laser doppler experimental protocol to measure capsaicin-induced dermal vasodilation (CIDV) and antagonist effect of peptide and small molecule antagonists. **(A)** Antagonist screening, dose ranging and dose-duration studies. **(B)** Onset study at 0 min.

Experimental design is outlined in Figure 1. Male and female C57BL/6J mice were recruited at 10–12 weeks of age at 20–30 g bodyweight. Animals were randomly allocated to each treatment group. Antagonists were diluted from respective stock solutions into 37°C pre-warmed sterile 0.9% saline containing 0.1% BSA and DMSO at a final amount of 3.2%. The vehicle was 0.9% saline supplemented with 0.1% BSA and 3.2% DMSO. Antagonist or vehicle was administered subcutaneously at a volume of 10 ml/kg. The anesthetic was: ketamine at 10 mg/ml and xylazine at 1 mg/ml, dissolved in sterile 0.9% saline. This was administered *via* the intraperitoneal route at 10 ml/kg and the anesthetized mouse placed onto a heating pad to maintain constant body temperature. The head was positioned for the dorsal region of both ears to be aligned to the LDI2-HIR Laser Doppler Imager (Moor Instruments) above it. The imager scanned 40 cm from the ear at a scan speed of 4 ms/pixel, with a scan area of approximately 11.0 cm × 4.1 cm and 256 × 45 pixels resolution. This provided a scan rate of approximately 1 scan/min for both ears. Both ears were simultaneously scanned for 3 min to generate the baseline blood flow prior to capsaicin-challenge (Figure 1). Guided by earlier research (Grant et al., 2002), capsaicin (Sigma-Aldrich, St. Louis, MO, United States) dissolved in absolute ethanol was applied topically to the ear (60 μg/ear; 10 μl on both dorsal and ventral side). On the contralateral ear, ethanol was applied as a negative control. Both ears were then immediately scanned with the Laser Doppler imager for a continuous 15-min period to capture changes in blood flow (Figure 1). The 15-min duration measurement period was chosen based on pilot trials and the literature (Grant et al., 2002), which indicated that a maximal and sustained increase in blood flow was achieved by this timepoint.

### Capsaicin-Induced Dermal Vasodilation – Antagonist Screening and Dose-Ranging Study

Mice were administered subcutaneously with vehicle or antagonist (Figure 1A). Ten minutes later mice were anesthetized and placed on a heating pad. Ten minutes after anesthesia induction, measurement of dermal blood-flow commenced. Either a single dose of antagonist as specified in the figures was used or a dose-ranging study was conducted. For the dose-ranging study, either hαCGRP_8-37_ or hαCGRP_8-37_ V8C-palmitate (V8C-palmitate) at a dose of 320, 960 or 3,200 nmol/kg was used. Doses were selected based on existing literature and our own pilot trials to characterize optimal (and maximal/minimal) effective dose within the bounds of solubility limits (Saxen et al., 1994; Grant et al., 2002; Gohin et al., 2015; Aubdool et al., 2016).

### Capsaicin-Induced Dermal Vasodilation - Dose-Duration Study

Mice were administered subcutaneously with vehicle or either hαCGRP_8-37_ (960 nmol/kg) or V8C-palmitate (960 nmol/kg) at 10, 20, 30 or 60 min prior to anesthesia and baseline read (Figure 1A). Ten minutes after anesthesia induction, measurement of dermal blood-flow began. To study the time of onset of antagonist activity, a shorter period between treatment administration and capsaicin challenge was utilized (T_0_) (Figure 1B). Here, mice were anesthetized and placed on the heating pad prior to peptide administration. Immediately after peptide administration, measurement of dermal blood-flow began.

### Data Analysis

Data were analyzed using GraphPad Prism versions 7–9 (GraphPad Software Inc., San Diego, CA, Unite States). Concentration-response cAMP data were fitted *via* non-linear regression using a four-parameter logistic equation. An extra sum-of-squares F-test was conducted to determine whether the Hill slope was equal to one. Where it was not significantly different to one, the data were fitted using a three-parameter logistic equation instead. An extra sum-of-squares F-test was also conducted to determine if two curves fitted onto two distinct datasets were significantly different from a single curve fit applied to both datasets to confirm if curve shifts are legitimate. The maximal (E_max_) and minimal (E_min_) responses were not constrained between independent experiments to obtain the pEC_50_ value.

For single concentrations of antagonist or global Schild analyses, the data were fitted to a concentration-response curve *via* non-linear regression using the Gaddum/Schild EC_50_ shift equation (Arunlakshana and Schild, 1958). The Hill slope was constrained to one following agonist analyses. The Schild slope was also constrained to one. The pA_2_ and pK_B_ values were obtained from the Schild analysis. For washout experiments, the same method of analysis was applied to the antagonists, but with minor revisions. The Schild analysis relied on the matched control agonist curves i.e. those derived from the same experimental conditions as the agonist + antagonist curves. Normalization was also specific to the matched control agonist curves.

Independent experiments were converted into a combined concentration-response graph by normalizing to control agonist E_max_ as 100% and control agonist E_min_ as 0%. Mean pEC_50_, pK_B_ and pA_2_ values from at least three independent experiments before normalization were also presented as mean ± SEM. pEC_50_, pK_B_ and pA_2_ values from independent experiments were grouped and compared by unpaired Student’s t-test or by one-way ANOVA. Alternatively, Student’s t-test comparisons between washout and no washout pA_2_ values of a particular antagonist were paired instead of unpaired, as the two conditions were always tested side-by-side.

For laser doppler imaging (LDI) scans, mean flux values of the whole scanned ear region for both ethanol (control) and capsaicin-treated ears at each time point (per minute) were analyzed using the MoorLDI Review 6.1 software. Mean flux values were normalized to the mean flux values averaged from the three continuous baseline scans and a time course is generated. Area under the curve (AUC) of the % flux mean after capsaicin application (t ≥ 0) was measured for each animal, grouped and compared between different ears or treatment arms. For statistical analysis and comparisons of treatment or sex, AUC values from different mice were grouped and compared using an unpaired Student’s t-test or by one-way ANOVA following a Shapiro-Wilk normality test. Time courses and time points were compared using repeated measures two-way ANOVA with Bonferonni’s multiple comparisons test.

## Results

**FIGURE 2:**
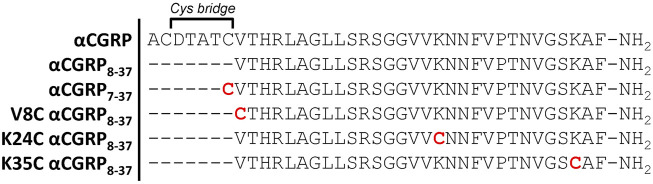
Amino acid sequences of human αCGRP_8-37_ and truncated cysteine-substituted peptide analogues. Red cysteine residues were subsequently modified by palmitoylation to yield the corresponding lipidated αCGRP_8-37_ analogues. αCGRP_7-37_ retains the native cysteine amino acid at position 7.

We first utilized the human αCGRP_8-37_ peptide backbone as a template to develop palmitoylated derivatives (Figure 2). CGRP possesses two native cysteine residues at amino acid positions 2 and 7, which are absent in αCGRP_8-37_. The synthesis route first required the synthesis of cysteine-substituted peptides (Figure 2) in order to provide a free thiol group for attachment of the palmitoyl moiety (Williams et al., 2018). We selected three positions on the αCGRP_8-37_ peptide backbone, Val-8, Lys-24, and Lys-35, for cysteine substitution sites and subsequent palmitoylation based on prior data that they could support modification without sacrificing binding affinity (Rist et al., 1999; Watkins et al., 2013; Booe et al., 2015; Liang et al., 2018). In addition, we selected the truncated peptide antagonist, αCGRP_7-37_, which retains the native Cys-7 residue for palmitoylation (Figure 2).

### αCGRP_7-37_ and Cysteine-Substituted αCGRP_8-37_ Analogues Retain Antagonist Activities at Human CGRP and AMY_1_ Receptors

αCGRP_8-37_ is the most characterized peptide antagonist at CGRP-responsive receptors (Hay et al., 2018). We therefore first validated hαCGRP_8-37_ as a reference antagonist with our human assay systems (Supplementary Table S1). Agonist stimulation by αCGRP in the presence or absence of αCGRP_8-37_ was performed in Cos-7 cells transfected with either CGRP or AMY_1_ receptors. As expected, αCGRP_8-37_ caused a rightward shift in the respective concentration-response curves with no discernible effects on maximal responses, allowing for pA_2_ values to be measured. The pEC_50_ and pA_2_ values derived for αCGRP agonism and hαCGRP_8-37_ antagonist activity (Supplementary Table S1) are consistent with literature values (Bailey and Hay, 2006; Hay et al., 2018). We then investigated antagonist activities of each cysteine-substituted analogue together with αCGRP_7-37_ at CGRP and AMY_1_ receptors. Each antagonist at a 30 nM concentration evoked a rightward shift in the respective agonist concentration-response curve, again with no significant change in maximal response at either CGRP (Supplementary Figure S1) and AMY_1_ receptor (Supplementary Figure S2). Compared to αCGRP_8-37_, K24C αCGRP_8-37_ and K35C αCGRP_8-37_ showed similar antagonist activities while αCGRP_7-37_ and V8C αCGRP_8-37_ were 4.5-fold and 25-fold less potent, respectively, at the CGRP receptor (Supplementary Table S1). At the AMY_1_ receptor, only V8C αCGRP_8-37_ displayed reduced antagonist activity compared to αCGRP_8-37_ (Supplementary Table S1).

### Palmitoylation at the N-Terminus or at Position 24 but not at the C-Terminal Region (Position 35) Retains Antagonist Activities Comparable to αCGRP_8-37_ at CGRP and AMY_1_ Receptors

**FIGURE 3:**
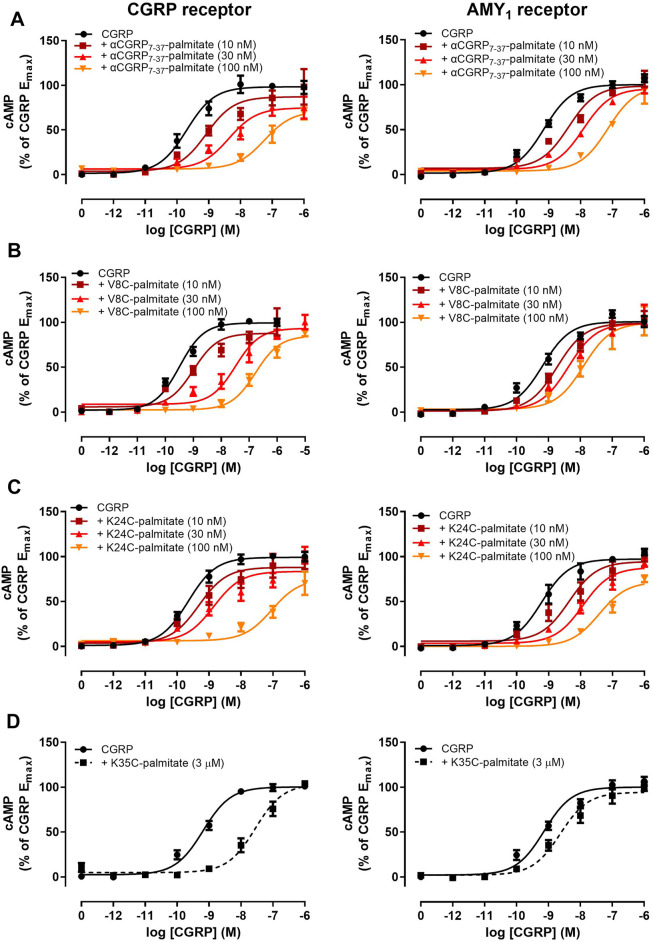
Antagonism of CGRP-stimulated cAMP production by lipidated αCGRP_8-37_ analogues at human CGRP or AMY_1_ receptors expressed in Cos-7 cells. Concentration-response curves were generated in the absence or presence of **(A)** αCGRP_7-37_-palmitate, **(B)** V8C-palmitate, **(C)** K24C-palmitate, and **(D)** K35C-palmitate at one or multiple different concentrations. Data points are plotted as a percentage of maximal CGRP-stimulated cAMP production as mean ± SEM of four to five independent experiments.

We next proceeded to investigate the antagonist activities of the respective palmitoylated peptides (Figure 3). Pilot experiments with a single 30 nM concentration of each lipidated hαCGRP_8-37_ analogue revealed apparent differences in antagonist activities and effects on agonist maximal response (data not shown). Therefore, full Schild analyses were conducted. These experiments confirmed that hαCGRP_8-37_ as the reference antagonist displayed competitive antagonism at both human CGRP and AMY_1_ receptors (Figure 3). Similarly, all lipidated hαCGRP_8-37_ analogues showed competitive antagonist behavior (Figure 3) with derived pK_B_ values shown in Table 1. Attachment of the palmitoyl moiety at the N-terminus (hαCGRP_7-37_-palmitate and hαCGRP_8-37_ V8C-palmitate; V8C-palmitate) or at position 24 (hαCGRP_8-37_ K24C-palmitate; K24C-palmitate) of hαCGRP_8-37_ had little effect on antagonist activity as compared to hαCGRP_8-37_ at the CGRP receptor (Figures 3A–C and Table 1). At the AMY_1_ receptor, these lipidated peptides displayed significantly stronger antagonist activity than hαCGRP_8-37_ (Figures 3A–C and Table 1). Interestingly, attachment of the palmitoyl moiety near the C-terminus of hαCGRP_8-37_ (hαCGRP_8-37_ K35C-palmitate; K35C-palmitate) substantially decreased antagonist activity as compared to hαCGRP_8-37_ at CGRP (∼70-fold reduction) and AMY_1_ receptors (∼12-fold reduction) (Figure 3D and Table 1).

**TABLE 1:** Antagonist activities of lipidated αCGRP_8-37_ analogues at calcitonin-family receptors expressed in Cos-7 cells or SK-N-MC cells with endogenous CGRP receptor expression.

	αCGRP_8-37_	αCGRP_7-37_-palmitate	V8C-palmitate	K24C-palmitate	K35C-palmitate
CGRPr (pK_B_)	9.09 ± 0.16 (5)	8.78 ± 0.06 (4)	9.35 ± 0.17 (5)	8.58 ± 0.25 (5)	7.24 ± 0.20 (5)^a^
AMY_1_r (pK_B_)	7.12 ± 0.13 (5)	8.80 ± 0.18 (5)^a^	8.26 ± 0.21 (5)^a^	8.75 ± 0.10 (5)^a^	6.01 ± 0.12 (5)^a^
AM_1_r (pA_2_)	7.36 ± 0.11 (9)	8.53 ± 0.17 (5)^a^	8.73 ± 0.17 (5)^a^	9.23 ± 0.16 (5)^a^	6.30 ± 0.14 (5)^a^
AM_2_r (pA_2_)	7.32 ± 0.20 (9)	8.55 ± 0.16 (5)^a^	8.18 ± 0.13 (5)^b^	9.18 ± 0.18 (5)^a^	6.60 ± 0.21 (5)^b^
CTr (pA_2_)	—^c^	7.11 ± 0.10 (5)	6.85 ± 0.13 (5)	6.21 ± 0.19 (5)	5.39 ± 0.30 (3)^d^
SK-N-MC (pA_2_)	9.58 ± 0.15 (6)	10.50 ± 0.19 (5)^e^	10.45 ± 0.24 (5)^e^	10.14 ± 0.17 (5)	7.78 ± 0.25 (5)^a^

Values are mean ± SEM of (*n*) independent experiments. pK_B_ or pA_2_ comparisons to αCGRP_8-37_ were analyzed by one-way ANOVA followed by Dunnett’s multiple comparison test.

a *p* < 0.001.

b *p* < 0.05

c αCGRP_8-37_ was too weak to generate a pA2 in the pilot experiments and sCT_8-32_ was used as a positive control for the CT receptor.

d Denotes that five experiments were conducted but only (n) repeats elicited measurable pA_2_ values.

e *p* < 0.01

### Lipidated αCGRP_8-37_ Analogues Display Enhanced Antagonist Activities at the Human CGRP Receptor Endogenously Expressed in SK-N-MC Cells

**FIGURE 4:**
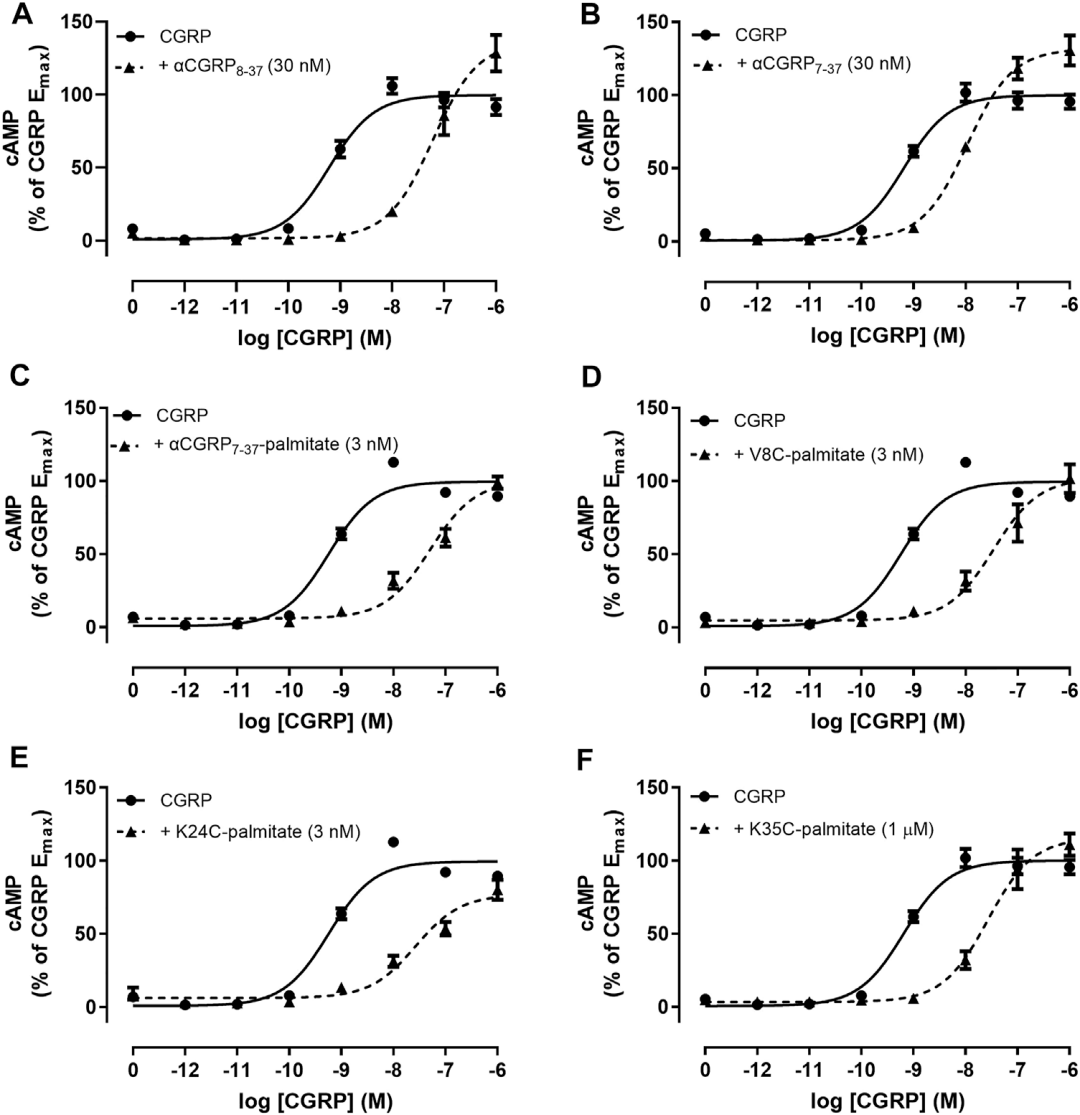
Antagonism by αCGRP_8-37_, αCGRP_7-37_, and lipidated analogues in SK-N-MC cells. CGRP concentration-response curves were generated in the absence or presence of **(A)** αCGRP_8-37_, **(B)** αCGRP_7-37_, **(C)** αCGRP_7-37_-palmitate, **(D)** V8C-palmitate, **(E)** K24C-palmitate, or **(F)** K35C-palmitate. Data points are plotted as a percentage of maximal CGRP-stimulated cAMP production with mean ± SEM of five to six independent experiments.

We measured antagonist activities in human SK-N-MC cells which endogenously express the CGRP receptor (Choksi et al., 2002). The αCGRP agonist pEC_50_ in SK-N-MC cells was 9.26 ± 0.05 (*n* = 13). The derived pA_2_ value for hαCGRP_8-37_ antagonist activity in SK-N-MC cells was similar to the pK_B_ value calculated in Cos-7 cells expressing the CGRP receptor (Table 1). Similar to the findings with transient CGRP receptor expression, all lipidated hαCGRP_8-37_ analogues displayed measurable competitive antagonist activity at a single concentration with no observable decrease in maximal response (Figure 4). Interestingly, compared to hαCGRP_8-37_, the derived pA_2_ values for αCGRP_7-37_-palmitate and V8C-palmitate were significantly higher than hαCGRP_8-37_ demonstrating increased antagonist activity (Table 1). Consistent with data from transient CGRP receptor expression, the derived pA_2_ for K35C-palmitate was significantly lower than hαCGRP_8-37_ (Table 1).

### Lipidated αCGRP_8-37_ Analogues but not αCGRP_8-37_ Exhibit Behavior Consistent With Delayed Dissociation at CGRP and AMY_1_ Receptors

As addition of a lipid moiety could affect membrane or receptor residence time (Ray et al., 2017; Fletcher et al., 2021) we investigated whether pre-incubation and wash-out of either hαCGRP_8-37_ or lipidated peptides with either CGRP and AMY_1_ receptors transfected in Cos-7 cells affected the observed pharmacology. For these experiments, the peptide antagonist was pre-incubated with cells for 15 min prior to agonist stimulation by αCGRP.

**FIGURE 5:**
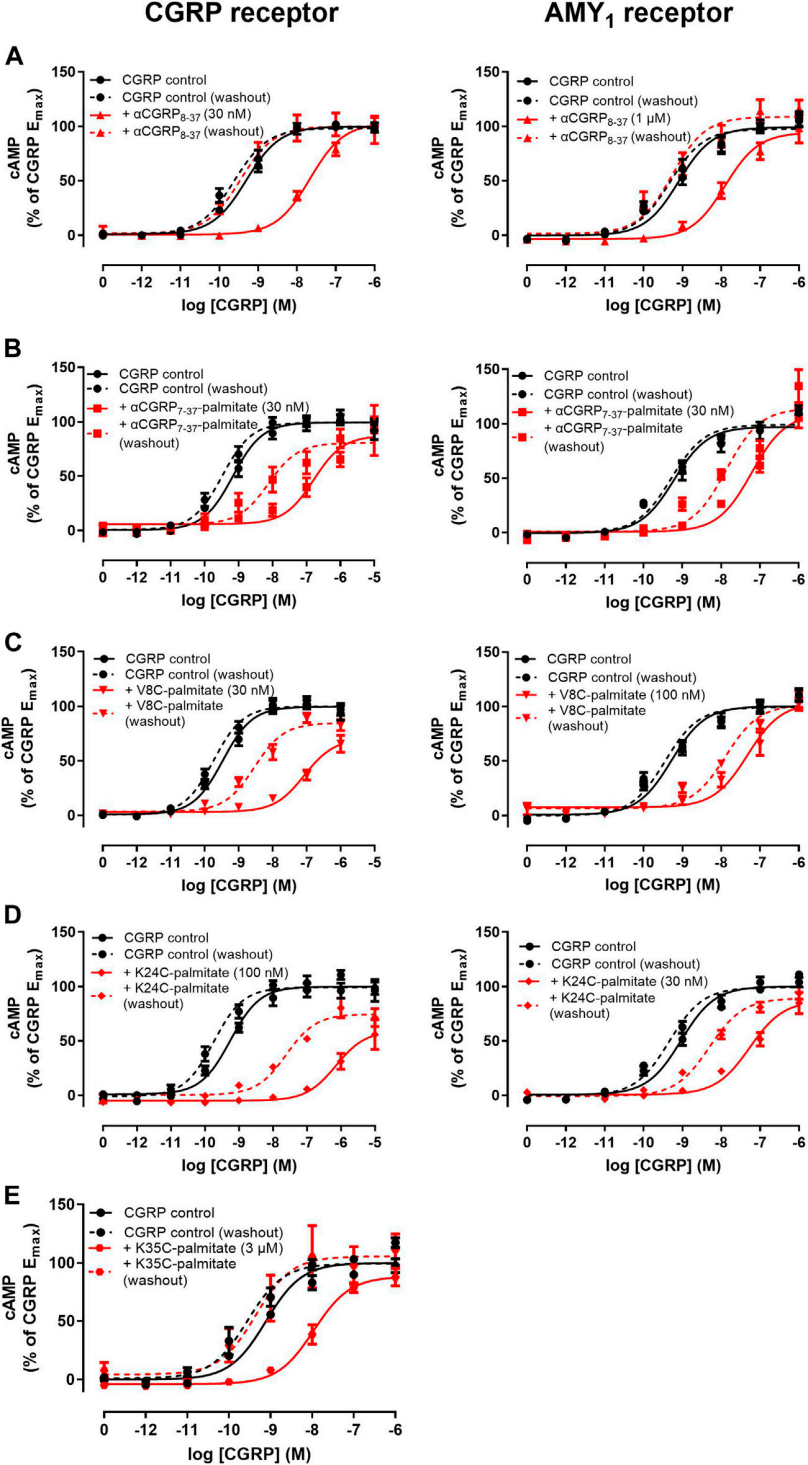
Effect of antagonist with pre-incubation and a washout step on agonist concentration-response curves at human CGRP and AMY_1_ receptors expressed in Cos-7 cells. **(A)** αCGRP_8-37_, **(B)** αCGRP_7-37_-palmitate, **(C)** V8C-palmitate, **(D)** K24C-palmitate, **(E)** K35C-palmitate. Solid and dashed lines indicate agonist concentration-response curves performed in the absence or presence of a washout step prior to agonist stimulation. Data points are plotted as a percentage of maximal CGRP-stimulated cAMP production and show mean ± SEM of five independent experiments.

Interestingly, we identified altered pharmacological behaviors unique to αCGRP_7-37_-palmitate, V8C-palmitate, and K24C-palmitate but not for K35C-palmitate or hαCGRP_8-37_ (Figure 5). Consistent with previous experiments, hαCGRP_8-37_ displayed similar antagonist activity under these experimental conditions (Figure 5A, solid red versus black lines; Table 2). However, the addition of a 15 min pre-incubation step for either αCGRP_7-37_-palmitate (Figure 5B), V8C-palmitate (Figure 5C), or K24C-palmitate (Figure 5D) resulted in increased antagonist activity at CGRP and AMY_1_ receptors compared to previously measured pK_B_ values (Table 2 versus Table 1).

**TABLE 2:** Effect of antagonist with pre-incubation and a washout step on pA_2_ values for lipidated αCGRP_8-37_ analogues at hCGRP and hAMY_1_ receptors expressed in Cos-7 cells.

		αCGRP_8-37_	αCGRP_7-37_-palmitate	V8C-palmitate	K24C-palmitate	K35C-palmitate
CGRPr	No washout	9.21 ± 0.21	9.86 ± 0.14	9.85 ± 0.08	10.28 ± 0.12	6.74 ± 0.16
	Washout	<7.5	9.34 ± 0.28^a^	8.67 ± 0.20^b^	9.13 ± 0.14^c^	<5.5^d^
AMY_1_r	No washout	7.23 ± 0.10	9.39 ± 0.22	8.98 ± 0.25	9.43 ± 0.20	—
	Washout	<6	8.69 ± 0.27	8.54 ± 0.31^a^	8.45 ± 0.23^a^	—

Values are mean ± SEM of five independent experiments. Comparisons between no washout and washout conditions were analyzed by paired Student’s t-test.

a *p* < 0.05.

b *p* < 0.001.

c *p* < 0.01.

d Denotes that only two out of five experiments elicited measurable pA_2_ values, averaging 5.60 ± 0.04.

Most notably, with the exception of K35C-palmitate, significant antagonist activity remained when a washout step was incorporated prior to agonist stimulation (Figures 5A–E, dashed red versus black lines). For comparison, the corresponding control agonist in each case also incorporated a wash out step comprising agonist stimulation performed in the absence of antagonist (dashed black line). Despite discernible and persistent antagonism, there was a reduction in pA_2_ values for V8C-palmitate and K24C-palmitate at both CGRP and AMY_1_ receptors. αCGRP_7-37_-palmitate only had reduction at the CGRP receptor (Table 2). By comparison, no retention of antagonist activity occurred for hαCGRP_8-37_ when a washout step was included at CGRP and AMY_1_ receptors (Figure 4A). These findings suggest that the palmitoyl moiety may delay peptide dissociation from the receptor or membrane.

### Lipidated αCGRP_8-37_ Analogues Display Antagonist Activities at AM_1_, AM_2_, and CT Receptors

Given the pharmacological overlap between the calcitonin family receptors (Hay et al., 2018), we also investigated antagonism by lipidated αCGRP_8-37_ analogues at AM_1_, AM_2_, and CT receptors. Receptor identities were confirmed pharmacologically with control antagonists (Table 1 and Supplementary Figure S3). In all cases, antagonist activity was observed at a single antagonist concentration (Table 1 and Supplementary Figure S4). At the AM_1_ receptor, all lipidated hαCGRP_8-37_ analogues with the exception of K35C-palmitate displayed higher antagonist activities compared to αCGRP_8-37_ (Table 1). Likewise, αCGRP_7-37_-palmitate and K24C-palmitate displayed increased antagonist activity at the AM_2_ receptor (Table 1). Consistent with previous findings, derived pA_2_ values for K35C-palmitate were significantly lower than αCGRP_8-37_ at both AM_1_ and AM_2_ receptors (Table 1). At the CTR, the lipidated hαCGRP_8-37_ analogues displayed measurable but otherwise weak antagonist activity (Table 1).

### Lipidated αCGRP_8-37_ Analogues Display Comparable Antagonist Activities Between Human and Mouse CGRP and AMY_1_ Receptors

Antagonist activities of lipidated hαCGRP_8-37_ analogues were also tested at mCGRP and AMY_1_ receptors as a bridge to *in vivo* studies in mice. The control agonist in this case, mαCGRP, displayed a potency of 9.59 ± 0.22 (*n* = 5) at the mCGRP receptor, and 7.82 ± 0.11 (*n* = 5) at the mAMY_1_ receptor, which was slightly lower than the mαCGRP pEC_50_ in Garelja et al. (2021). The comparatively lower potency of mαCGRP at the mAMY_1_ receptor versus the CGRP receptor is different to hαCGRP which is equipotent at both human receptors. However, there is some variation in the potency of mαCGRP at this receptor (Bohn et al., 2017). All lipidated αCGRP_8-37_ analogues displayed antagonist activity at the mCGRP receptor (Table 3 and Supplementary Figure S5). At the mAMY_1_ receptor, it was difficult to derive measurable pA_2_ values for the lipidated hαCGRP_8-37_ analogues due to the relatively low potency of the mCGRP peptide, however antagonism was observed for most of the peptides (Table 3 and Supplementary Figure S5).

**TABLE 3:** pA_2_ values for αCGRP_8-37_ and lipidated αCGRP_8-37_ analogues at mCGRP and AMY_1_ receptors expressed in Cos-7 cells.

	αCGRP_8-37_	αCGRP_7-37_-palmitate	V8C-palmitate	K24C-palmitate	K35C-palmitate
mCGRPr	9.00 ± 0.40 (5)	9.89 ± 0.35 (5)	9.70 ± 0.40 (4)^a^	9.03 ± 1.00 (3)^a^	7.56 ± 0.18 (4)^a^
mAMY_1_r	7.61 ± 0.93 (3)^a^	7.72 ± 0.28 (4)^a^	7.61 ± 0.20 (5)	<6 (3)^b^	6.78 ± 0.14 (3)^a^

Comparisons to αCGRP_8-37_ were analyzed by one-way ANOVA followed by Dunnett’s multiple comparison test. Values are mean ± SEM (*n*).

a Denotes that five experiments were conducted but only (n) repeats elicited measurable pA_2_ values.

b Two repeats elicited pA_2_ values of 7.92 and 5.93.

### Effects of Antagonists on the Capsaicin-Induced Dermal Vasodilatory Response

#### Establishing the Capsaicin-Induced Dermal Vasodilatory Model

**FIGURE 6:**
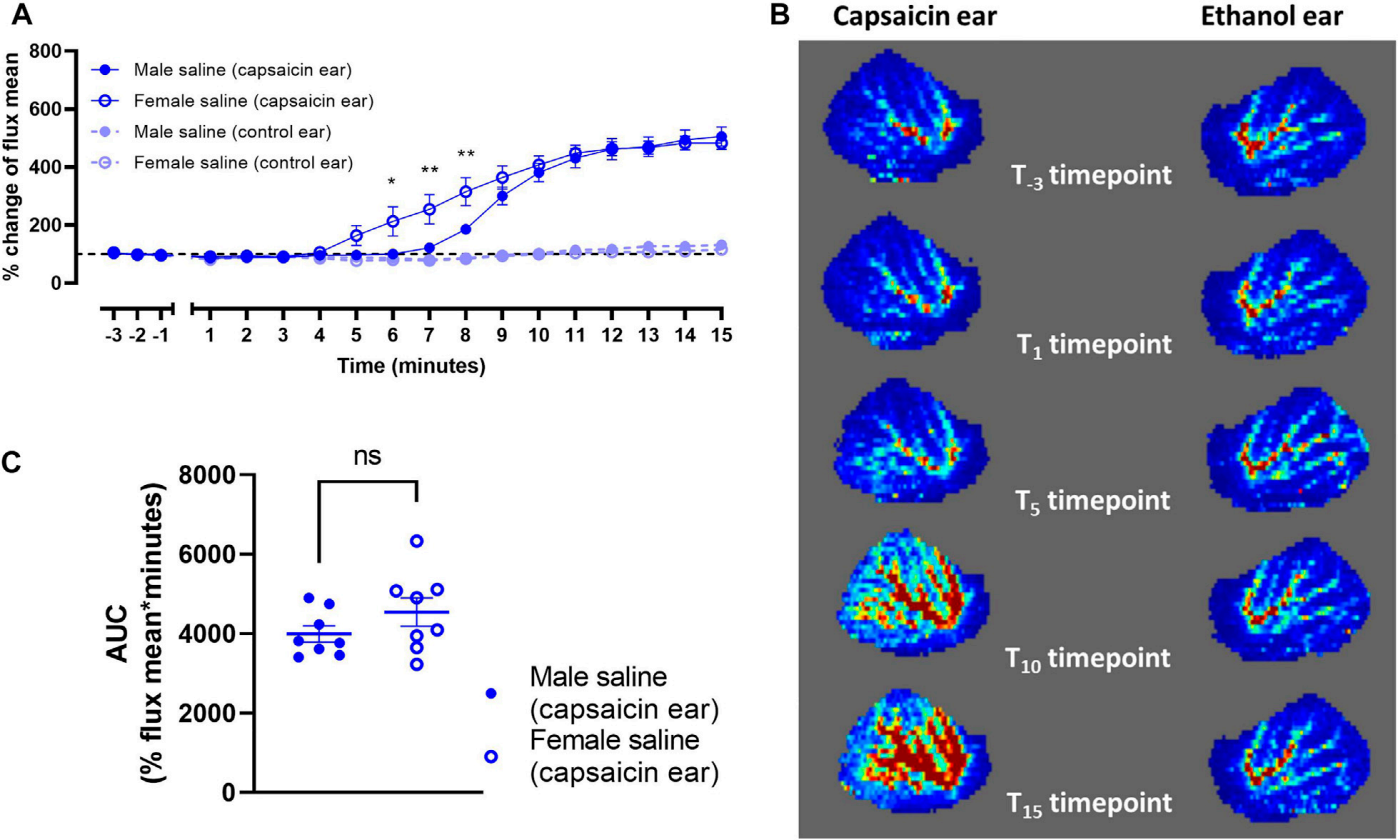
Capsaicin-induced dermal vasodilation in mouse ears of saline-treated male (*n* = 8) and female mice (*n* = 8). **(A)** Vasodilatory responses of ears following application of either capsaicin or ethanol (control). **(B)** Representative laser doppler flux images captured over the 15 min time course. **(C)** Corresponding area-under-curve (AUC) mean values over the 15 min time course for capsaicin-applied ears of male and female mice. Time points are marked where data points differ significantly between sex. ns *p* > 0.05, *p < 0.05, **p < 0.01.

Male and female mice were recruited to measure blood flow as an effect of topical capsaicin. Vehicle was injected subcutaneously followed by induction of anesthesia after 10 min. Capsaicin (in ethanol) evoked a robust increase in dermal blood flow over baseline by approximately five-fold over the 15 min measurement window (Figures 6A,B). By comparison, ethanol alone applied to the contralateral ear at the same time yielded no discernible vasodilatory response (Figures 6A,B). The rate of increase in vasodilatory responses between male and female mice diverged over the first ∼8 min (Figure 6A). However, both groups reached the same maximum response, and the overall absolute vasodilatory response was not significantly different as measured by AUC (Figure 6C). It is noted that female mice generated greater variability in vasodilatory response.

#### Lipidated hαCGRP_8-37_ Analogues Antagonize CGRP Action *in vivo*

**FIGURE 7:**
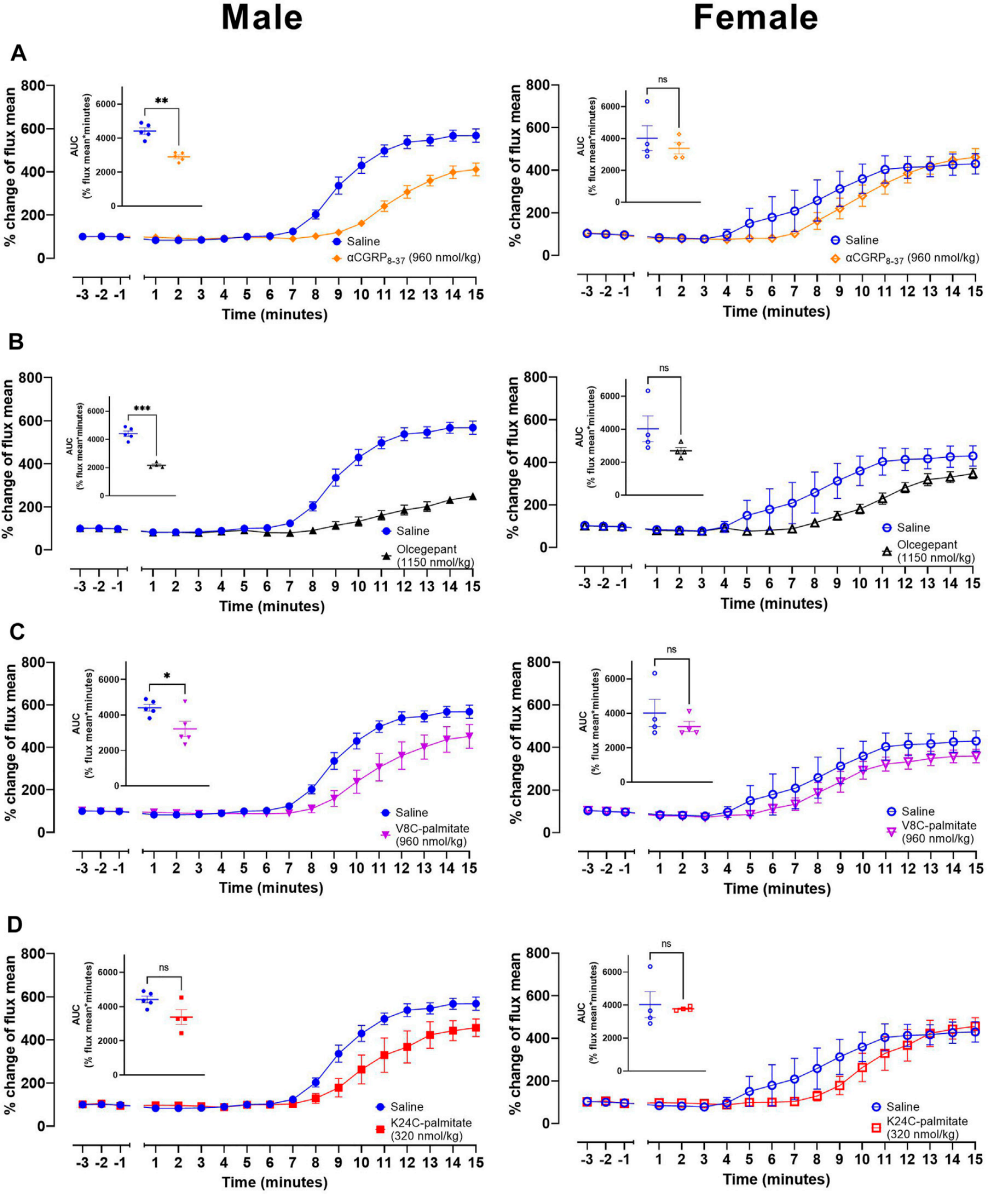
CGRP receptor antagonists attenuate CGRP action *in vivo*. Effects of **(A)** αCGRP_8-37_, **(B)** olcegepant, **(C)** V8C-palmitate and **(D)** K24C-palmitate on CIDV in male (left) and female (right) mice. Inset graphs show corresponding AUC mean values over the 15 min measurement timeframe following capsaicin application to the ear. ns *p* > 0.05, * *p* < 0.05, ** *p* < 0.01, *** *p* < 0.001. Each treatment group comprises four to five mice with corresponding saline controls.

Using these conditions we then undertook an investigation of the effect of antagonists on capsaicin-induced dermal vasodilatory (CIDV). Peptide antagonists were tested as well as the small molecule CGRP antagonist, BIBN4096BS (olcegepant) as an additional control. These exploratory studies were also conducted in male and female mice to explore if sex bias is present with respect to their effects on CIDV. Antagonist or vehicle was injected subcutaneously followed by induction of anesthesia after 10 min. For these experiments we used hαCGRP_8-37_ (Figure 7A), olcegepant (Figure 7B), V8C-palmitate (Figure 7C), and K24C-palmitate (Figure 7D). The K35C-palmitate peptide was not used due to its decreased receptor antagonism. The maximum dose that we could use for K24C-palmitate was 320 nmol/kg based on its limited solubility at high concentrations. All four antagonists attenuated the CIDV response (representative LDI scans shown in Supplementary Figures S6), but the effect was more pronounced in male versus female mice as shown by the relative time courses and mean AUC values between saline and antagonist-treated groups (Figure 7). Additionally, examination of the raw data indicated that there was no effect on basal blood flow by administration of saline or any of the antagonists.

In parallel, we also developed two further lipidated antagonists. These were R11C-palmitate and βV8C-palmitate. They were tested *in vitro* (Supplementary Figure S7) and then in the CIDV model, together with βCGRP_8-37_. These, and subsequent CIDV experiments, were only conducted in male mice due to the apparently greater variance in vasodilatory response in female mice and because our intent was to investigate target engagement which could be achieved from the males. αCGRP_7-37_-palmitate (Supplementary Figure S8A), R11C-palmitate (Supplementary Figure S8B), βCGRP_8-37_ (Supplementary Figure S8C), and βV8C-palmitate (Supplementary Figure S8D) were administered at a lower dose (mass-matched dosage) to prior experiments. Compared to V8C-palmitate and K24C-palmitate (Figure 7), these peptides displayed weaker antagonist activities versus the matched vehicle group.

**FIGURE 8:**
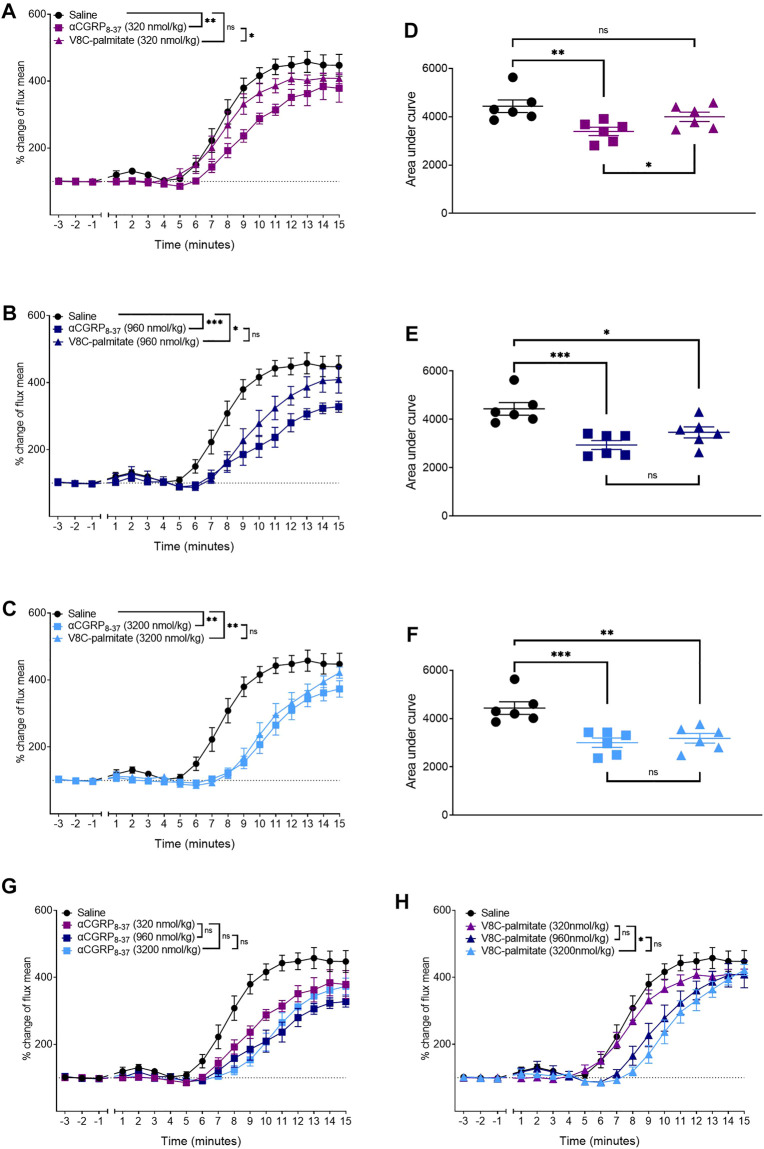
Dose-dependent effects of αCGRP_8-37_ and V8C-palmitate on CIDV in male mice. Peptides were administered at doses of 320 nmol/kg **(A,D)**, 960 nmol/kg, **(B,E)** or 3,200 nmol/kg **(C,F)**. Combined data for αCGRP_8-37_ and V8C-palmitate are shown in **(G,H)**, respectively. ns *p* > 0.05, *p < 0.05, **p < 0.01, ***p < 0.001. Each treatment group comprised six mice with corresponding saline controls.

Dose-ranging experiments were next conducted with V8C-palmitate, at 320, 960, and 3,200 nmol/kg (Figure 8). For comparison, hαCGRP_8-37_, was the reference antagonist at molar-matched dosages (Figure 8). V8C-palmitate did not have a significant antagonist effect on CIDV at the lowest administered does of 320 nmol/kg (Figures 8A,D). However, at 960 nmol/kg, V8C-palmitate significantly reduced CIDV, comparable to hαCGRP_8-37_ (Figures 8B,E). At the highest dose of 3,200 nmol/kg, V8C-palmitate again had a significant antagonist effect, comparable to that evoked by hαCGRP_8-37_ (Figures 8C,F). All three doses for both peptides are compared in Figures 8G,H.

**FIGURE 9:**
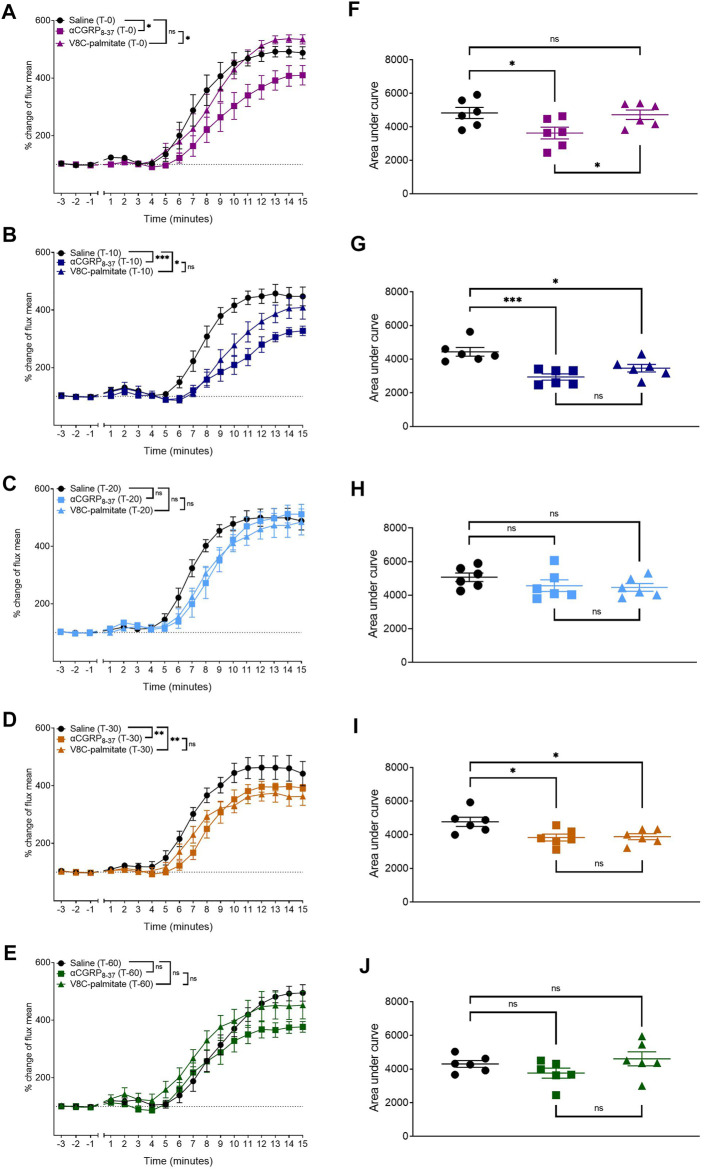
Comparison between αCGRP_8-37_ and V8C-palmitate for antagonism onset of CGRP action *in vivo*. αCGRP_8-37_ (960 nmol/kg) and V8C-palmitate (960 nmol/kg) were administered at 0 min **(A,F)**, 10 min **(B,G)**, 20 min **(C,H)**, 30 min **(D,I)** and 60 min **(E,J)** prior to anesthesia and capsaicin application. ns *p* > 0.05, *p < 0.05, **p < 0.01, ***p < 0.001. Each treatment group comprised six mice with corresponding saline controls.

To determine whether there were any temporal differences between the behavior of these antagonists, hαCGRP_8-37_ and V8C-palmitate were each administered at a dose of 960 nmol/kg, at T0, T-10, T-20, T-30, and T-60 timepoints prior to anesthesia and capsaicin application (Figure 9). hαCGRP_8-37_ attenuated the CIDV response immediately at the T0 time point (Figures 9A,F) after which antagonist activity waned completely by the T-60 time point (Figures 9E,J). By comparison, V8C-palmitate displayed a delayed effect on the CIDV response with attenuation evident only at T-10 (Figures 9B,G) before again disappearing by the T-60 timepoint (Figures 9E,J).

## Discussion

### Lipidated hαCGRP_8-37_ Analogues Retain Antagonist Activity *In Vitro*

Our findings show that it is possible to attach a palmitoyl moiety at selected positions of the CGRP_8-37_ peptide backbone and still preserve antagonist activity. We initially generated hαCGRP_8-37_ cysteine-substituted analogues to assess the effect of cystine-substitution at these residues prior to palmitoylation. The findings suggested that these residues were somewhat amenable to modification with cysteine. However, cysteine-substitution had only limited utility in predicting the effect of cysteine-lipidation (palmitoylation) at the same residues on hαCGRP_8-37_ pharmacology. Except for position 35, the resulting lipidation at cysteine positions 7, 8, and 24, generated analogues that retained antagonist activity at human CGRP and AMY_1_ receptors and at the human CGRP receptor endogenously expressed in SK-N-MC cells. Our analyses indicated that antagonism of these lipidated hαCGRP_8-37_ analogues at the human CGRP and AMY_1_ receptor was competitive as the respective E_max_ values were not significantly different. For V8C-palmitate, apparent competitive antagonism was demonstrated directly by increasing the concentration of agonist by an additional log unit to enable full curves to be achieved (Figure 3B). Nevertheless, we cannot fully discount the possibility of non-surmountable antagonism by hαCGRP_7-37_-palmitate and K24C-palmitate due to the lipid moiety preventing full dissociation from the receptor. More detailed investigation such as receptor binding studies would be required to resolve the precise mode of antagonism.

We also noted differences in antagonist activity by the lipidated hαCGRP_8-37_ analogues between Cos7 cells expressing the CGRP receptor and SK-N-MC cells expressing endogenous CGRP receptors. We speculate this difference could be due to cellular features specific to each experimental system such as receptor expression and distribution on the cell surface or cell membrane composition affecting lipidated peptide kinetics. Evidence also suggests that antagonist potency can differ depending on the signaling pathway measured (Walker et al., 2018). Thus, if the CGRP receptor couples to a different protein complement of signaling pathways between transfected Cos7 cells and SK-N-MC cells, then measurement of only cAMP could explain the differences in antagonist activity.

The positional effect of palmitoylation can be interpreted by considering the two-domain model of class B G protein-coupled receptor peptide ligand binding and receptor activation, along with receptor structures (Hoare, 2005; Booe et al., 2015; Liang et al., 2018). The peptide C-terminus plays an important role in binding, whereas the N-terminus is crucial for receptor activation. Attachment of a lipid moiety to most positions was able to preserve antagonism, whereas attachment to position 35 in the C-terminus substantially reduced antagonism. Lys-35 faces outwards into unoccupied space when CGRP is bound to the CGRP receptor (Liang et al., 2018) suggesting it is amenable to modification. However, the attached palmitoyl moiety appears to obstruct the peptide from initiating proper contact and binding to the CGRP receptor binding pocket (Booe et al., 2018). The effect of palmitoylation was generally similar between receptors.

### Lipidated hαCGRP_8-37_ Peptide Antagonists May Have Altered Receptor Binding Kinetics

Lipidated peptide analogues, except for K35C-palmitate, displayed unique pharmacological characteristics. First, there was increased antagonism when lipidated peptide analogues were pre-incubated with transfected CGRP and AMY_1_ receptors for a 15-min period prior to stimulation with agonist. This was not observed with hαCGRP_8-37_ which suggests an effect specific to some lipidated peptides. Second, some antagonists retained activity following washing of the cells. These findings suggest that the palmitate moiety may alter the receptor or membrane residence time of the peptide antagonist, which could also potentially explain the observed improved antagonist activities of αCGRP_7-37_-palmitate, V8C-palmitate, and K24C-palmitate, at AM_1_ and AM_2_ receptors compared to hαCGRP_8-37_. A similar outcome was observed in the pharmacological characterization of lipidated amylin analogues (Fletcher et al., 2021), where an extended receptor residence time was correlated with their prolonged duration of action. Although receptor residency time and binding kinetics are not the sole contributor to the efficacy of an agonist, they may have a strong influence on antagonist activity. One potential mechanism by which this could occur is through compartmentalization of the lipidated peptide with the cell membrane. Membrane partitioning and membrane trafficking of lipidated proteins have been reported to influence protein activity (Zacharias et al., 2002; Ostrom and Insel, 2004; Ray et al., 2017). Potentially, the palmitoyl moeity facilitates association of the peptide with the cell membrane and increases its local concentration within the vicinity of the target membrane receptors to alter kinetics and receptor residence time, and improve their antagonist activity.

### Lipidated αCGRP_8-37_ Peptide Antagonists Attenuate CGRP Action *In Vivo*

We utilized a CIDV animal model to demonstrate *in vivo* target engagement by the lipidated peptide antagonists. The vasodilatory response within the ear following application of capsaicin was robust with minimal response in the ethanol-treated contralateral ear, which was used as an internal control. The magnitude of the CIDV response at the capsaicin dose used (60 μg/ear) is similar to that used in previous studies (Grant et al., 2002; Starr et al., 2008). We validated this experimental system by demonstrating attenuation of the CIDV response by the reference antagonists, hαCGRP_8-37_ and olcegepant, showing successful target engagement with the CGRP receptor *in vivo*.

Female mice appeared to show an earlier vasodilatory onset compared to male mice although the respective total integrated responses as analyzed by AUC were comparable. This observation is consistent with reported differences in CGRP activity and vascular responses between male and female mice (Lee et al., 2003; Peng et al., 2011). Female mice also display a greater endothelium-dependent vasodilatory response to acetylcholine (Zuloaga et al., 2014). It is possible that female mice respond physiologically to capsaicin differently due to differences in CGRP receptor or peptide expression. Alternatively, responses could be influenced by the oestrus cycle phase. There is evidence that hormonal variations as part of the oestrus cycle modulates TRPV1, which is the major cation channel responsible for the capsaicin-evoked vasodilatory response (Artero-Morales et al., 2018). Thus, hormonal levels could potentially modulate intrinsic vasodilatory responses through TRPV1 expression and activity, and subsequently, affect the release of neurotransmitters or neuropeptides, including CGRP. Additionally, CIDV is an indirect measurement of CGRP activity, since CGRP release is dependent upon TRPV1 activation. It is possible that other substances are involved in the CIDV response, which could influence the effect of CGRP antagonists on CIDV.

The cellular mechanisms of CGRP vasodilatory effect can primarily be divided into endothelium-independent vasodilation or endothelium-dependent vasodilation (Brain and Grant, 2004). This adds a layer of complexity as the literature suggests differences in tissue and species specificity for these two mechanisms (Sohn et al., 2020). It is therefore possible that the temporal and sex differences observed stem from differential CGRP activity. Sex-specific differences in CGRP receptor expression could also impact on CGRP action *in vivo*. The receptor component protein (RCP) expression, which is a component of the CGRP receptor signaling complex (Ji et al., 2019), as well as CLR and RAMP1 expression have been shown to differ between male and female rodents (Stucky et al., 2011). Our study did not account for oestrus cycling or potential differences in CGRP receptor expression, so these remain interesting parameters to explore for future vasodilatory studies involving CGRP.

Peptide lipidation is a useful tool for developing efficacious peptide therapeutics by increasing peptide half-life and decreasing elimination rate. Peptide lipidation as a strategy has been explored previously with CGRP. Here, a modified αCGRP analogue with an albumin binding fatty acid moiety showed protracted pharmacokinetic properties (Nilsson et al., 2016; Sheykhzade et al., 2018) and demonstrated positive utility in alleviating or reversing cardiovascular disease in rodents (Aubdool et al., 2017). Another study reported modification of αCGRP with a fatty acid-dibenzylcyclooctyne (DIBO) moiety at position 24, which improved its plasma stability (Demin, 2018).

By comparison, the current lipidation strategy produced the V8C-palmitate and K24C-palmitate analogues that attenuated CIDV response *in vivo*. We confirmed that the mouse model was an appropriate translational model as the antagonist activities of hαCGRP_8-37_ and the lipidated hαCGRP_8-37_ analogues were comparable across mouse and human CGRP receptors. Dose-ranging experiments showed that a higher dose of V8C-palmitate was required compared to hαCGRP_8-37_ to reach a similar attenuation of the CIDV response. Likewise, time to onset experiments indicated that V8C-palmitate displayed a longer onset of action compared to hαCGRP_8-37_, although attenuation of CIDV was lost after 60 min in both cases. These observations for V8C-palmitate could reflect a depot effect resulting in delayed drug absorption into the blood through the subcutaneous injection route or a slower distribution to the tissue region from plasma compared to hαCGRP_8-37_. This raises the possibility of sustained slow release of the V8C-palmitate analogue into the systemic circulation and/or site of action compared to hαCGRP_8-37_.

## Conclusion

hCGRP_8-37_ peptide analogues were palmitoylated at different locations on the peptide sequence. Excluding the C-terminally modified analogue, K35C-palmitate, the lipidated analogues behaved as competitive antagonists at the CGRP and AMY_1_ receptor *in vitro*. There was evidence that the palmitoyl moiety on the peptide antagonist confers altered residence time compared to hαCGRP_8-37_ as observed by increased antagonist activity with prolonged incubation of lipidated peptide analogues with CGRP and AMY_1_ receptors prior to agonist stimulation and retention of antagonist activity when a washout step was included prior to agonist stimulation.

For the translational studies in mice, V8C-palmitate and K24C-palmitate significantly attenuated the CIDV response, demonstrating successful target engagement of the CGRP receptor *in vivo*. However, there was a difference in dose-response profile and onset indicating the presence of a depot effect for the lipidated analogue. Overall, these findings show it is possible to generate palmitoylated peptides based on the hαCGRP_8-37_ peptide backbone that retain both antagonist activity at CGRP and AMY_1_ receptors, and attenuate CGRP action *in vivo*. Although dedicated pharmacokinetic studies are required, these findings suggest that lipidation may offer a route to develop a new class of CGRP peptide antagonists as therapeutics.

## Data Availability

The original contributions presented in the study are included in the article/Supplementary Material, further inquiries can be directed to the corresponding authors.
