# Supplementary material for: Lipidated Calcitonin Gene-Related Peptide (CGRP) Peptide Antagonists Retain CGRP Receptor Activity and Attenuate CGRP Action In Vivo
Source: Front Pharmacol. 2022 Mar 7;13:832589. doi: 10.3389/fphar.2022.832589 (PMC8942775; doi:10.3389/fphar.2022.832589)
Supplement: Supplementary file 1 [file DataSheet1.docx]

Supplementary Material

# Supplementary Figures and Tables

## Supplementary Figures

**
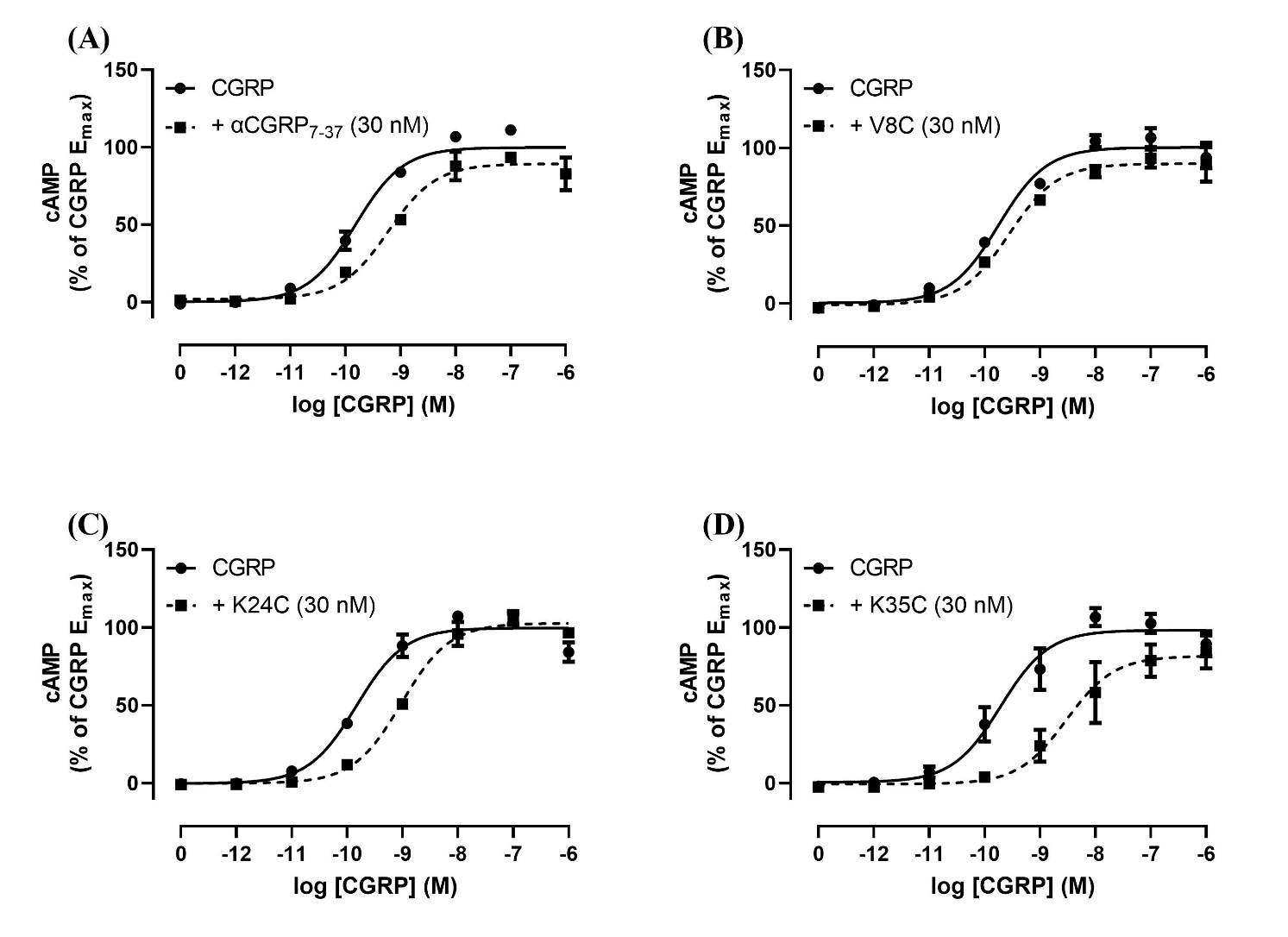
**

**Supplementary Figure 1.** Antagonist activity observed for (A) αCGRP_7-37_, (B) V8C, (C) K24C, (D) K35C at the hCGRP receptor expressed in Cos-7 cells. Data points were plotted as a percentage of maximal CGRP-stimulated cAMP production and shown as mean ± SEM of 3 independent experiments.


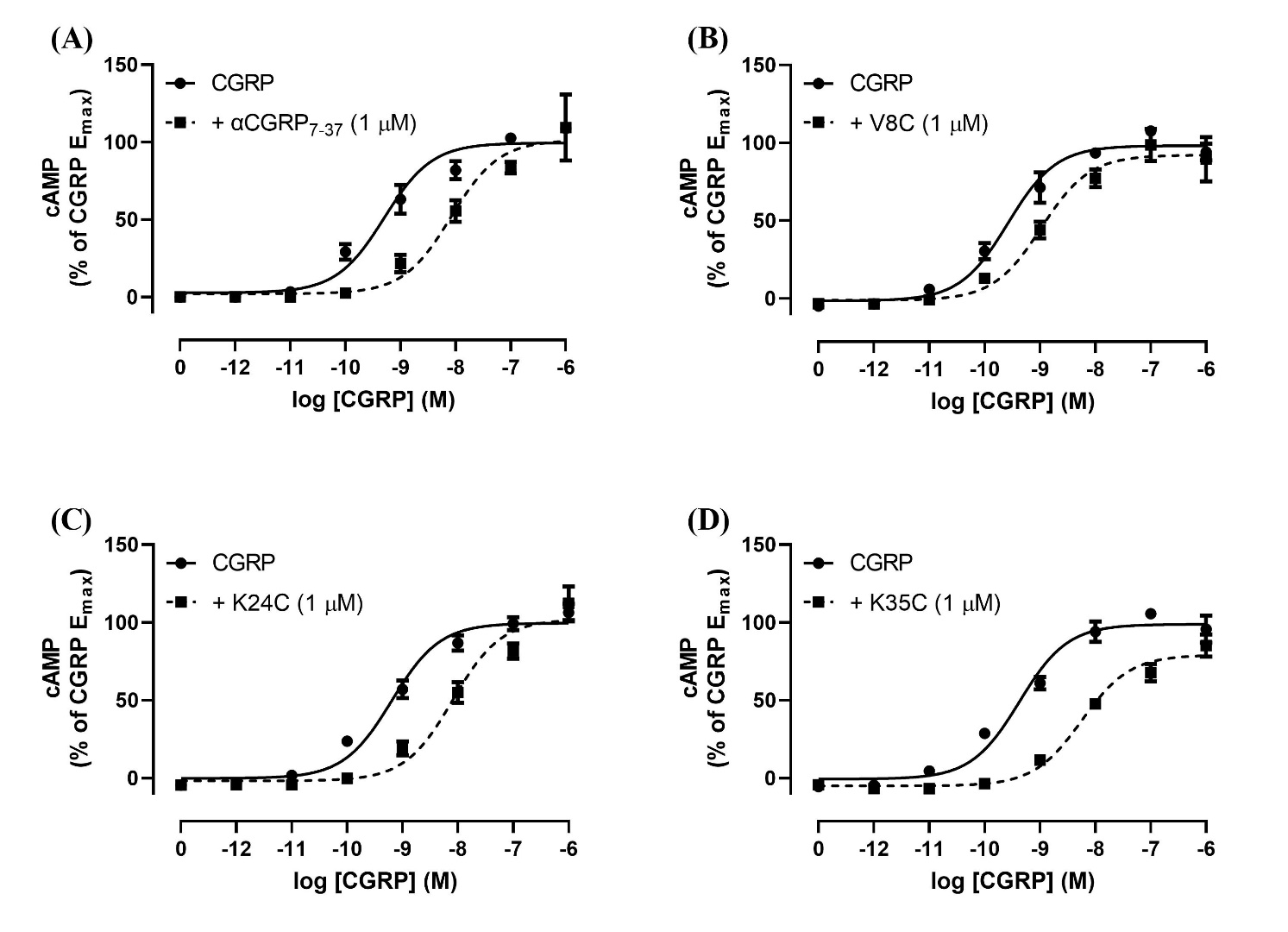


**Supplementary Figure 2.** Antagonist activity observed for (A) αCGRP_7-37_, (B) V8C, (C) K24C, (D) K35C at the hAMY_1_ receptor expressed in Cos-7 cells. Data points were plotted as a percentage of maximal CGRP-stimulated cAMP production and shown as mean ± SEM of 3 independent experiments.


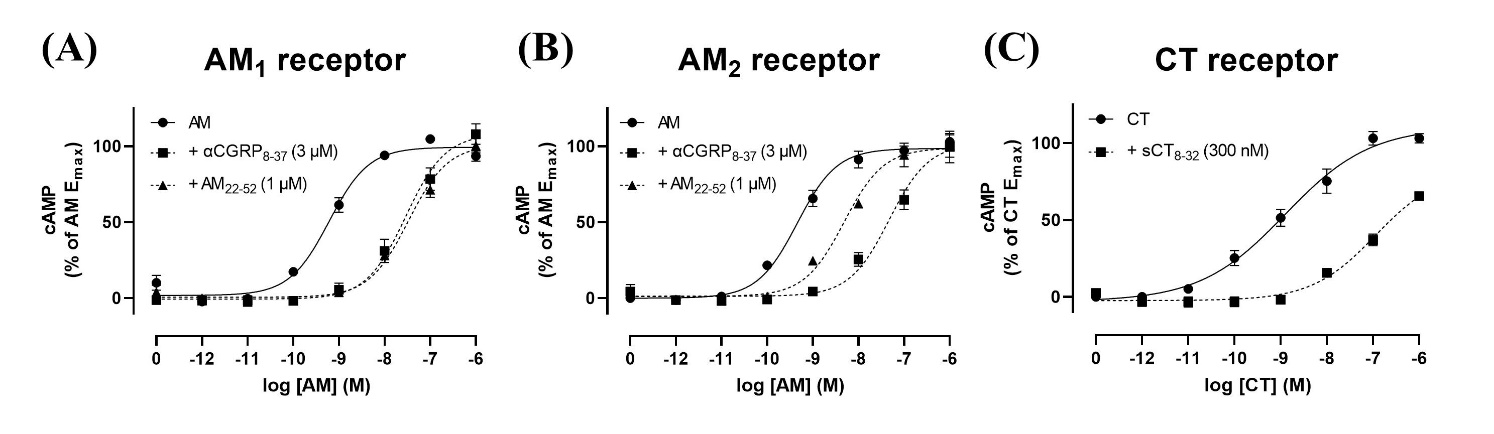


**Supplementary Figure 3.** Antagonist activity observed for the control antagonists αCGRP_8-37_ (n=9), AM_22-52_ (n=9), and sCT_8-32_ (n=6) at the (A) hAM_1_ receptor, (B) hAM_2_ receptor, and (C) hcalcitonin receptor (CTR) expressed in Cos-7 cells. Data points are plotted as a percentage of maximal AM- or calcitonin (CT)-stimulated cAMP production and show mean ± SEM of 5-9 independent experiments.


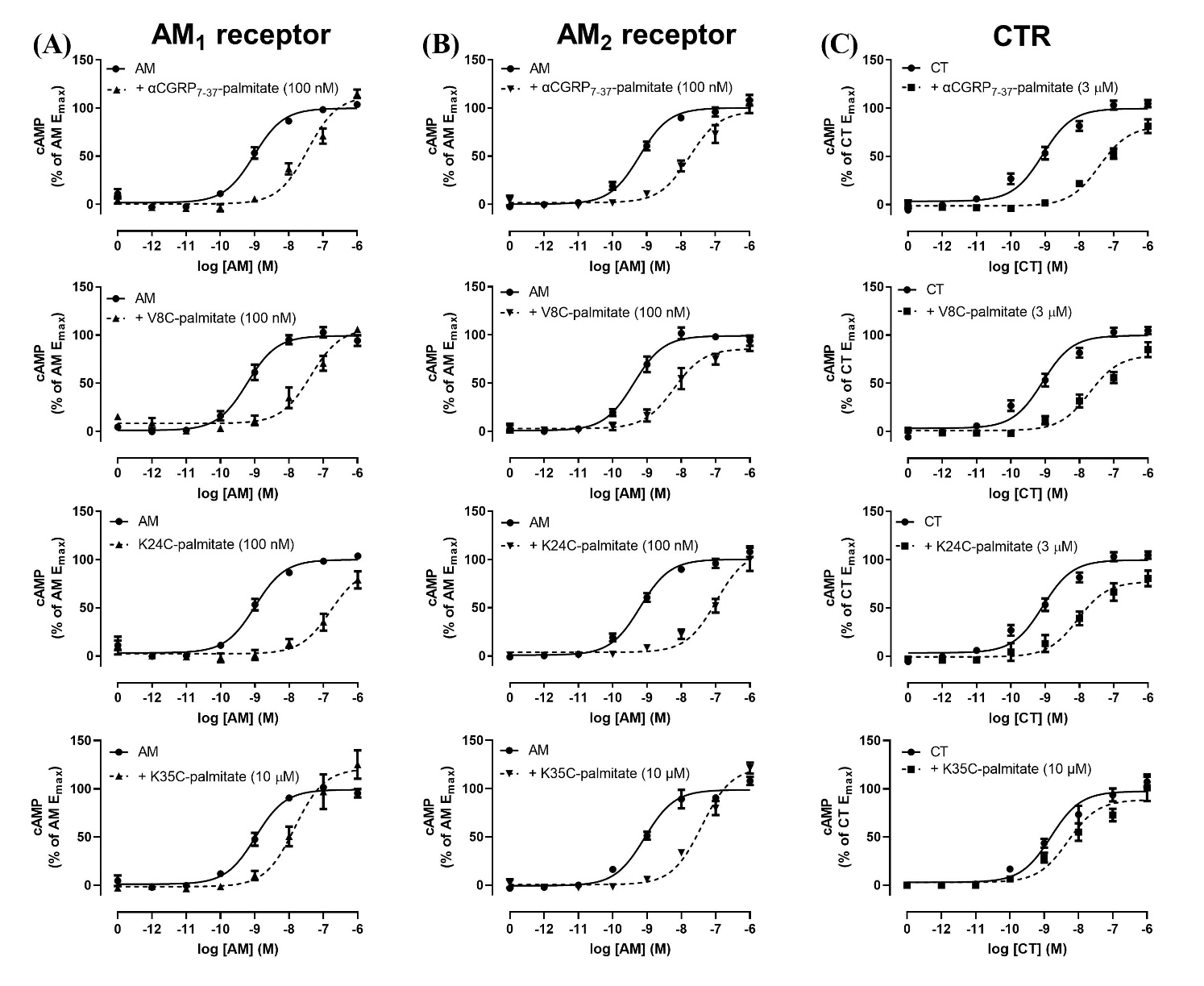


**Supplementary Figure 4.** Antagonist activity observed for the lipidated αCGRP_8-37_ analogues at the (A) hAM_1_ receptor, (B) hAM_2_ receptor, and (C) hcalcitonin receptor (CTR) expressed in Cos-7 cells. Data points are plotted as a percentage of maximal AM- or calcitonin (CT)-stimulated cAMP production and show mean ± SEM of 3-5 independent experiments.


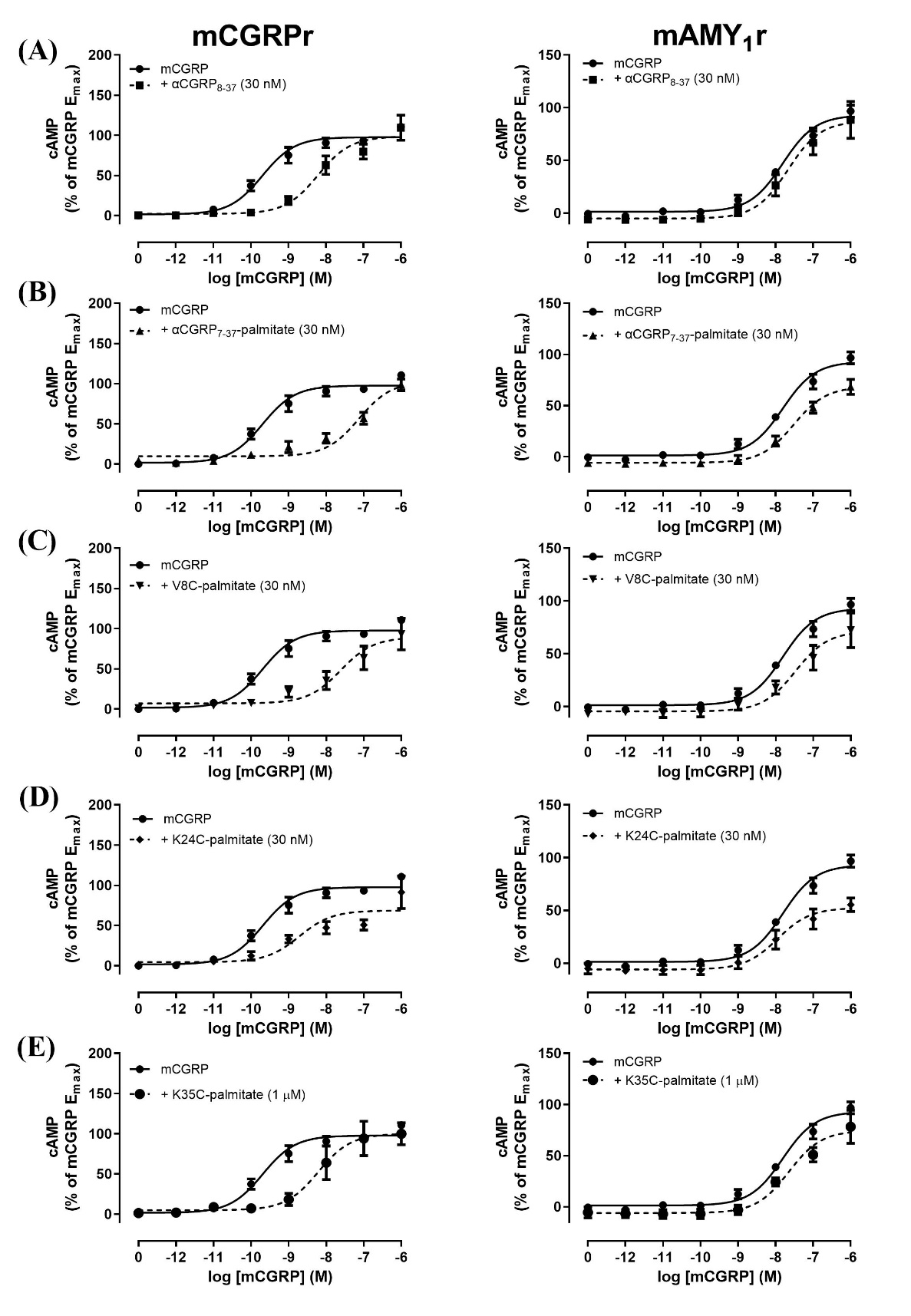


**Supplementary Figure 5.** Antagonism by lipidated αCGRP_8-37_ analogues at mCGRP or AMY_1_ receptors expressed in Cos-7 cells. CGRP concentration-response curves were conducted in the absence and presence of (A) αCGRP_8-37_, (B) αCGRP_7-37_-palmitate, (C) V8C-palmitate, (D) K24C-palmitate, and (E) K35C-palmitate at one or multiple different concentrations. Data points were plotted as a percentage of maximal CGRP-stimulated cAMP production and shown as mean ± SEM of 4-5 independent experiments.

**
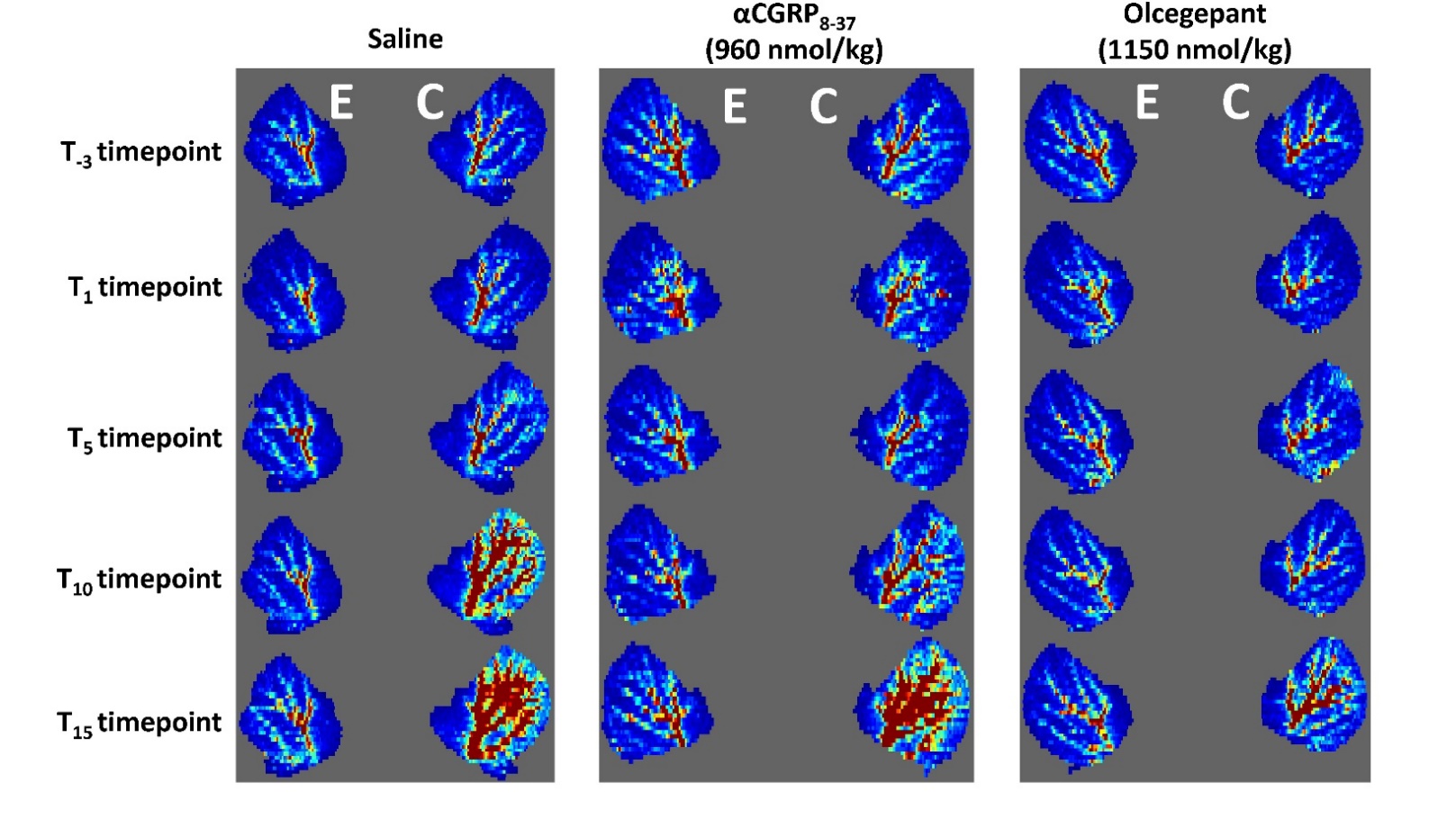
**

**Supplementary Figure 6.** Representative LDI scans at the various timepoints (T) for saline, αCGRP_8-37_ (960 nmol/kg), and olcegepant (1150 nmol/kg), showing the ethanol-treated (E) ear and the capsaicin-treated (C) contralateral ear of male mice.


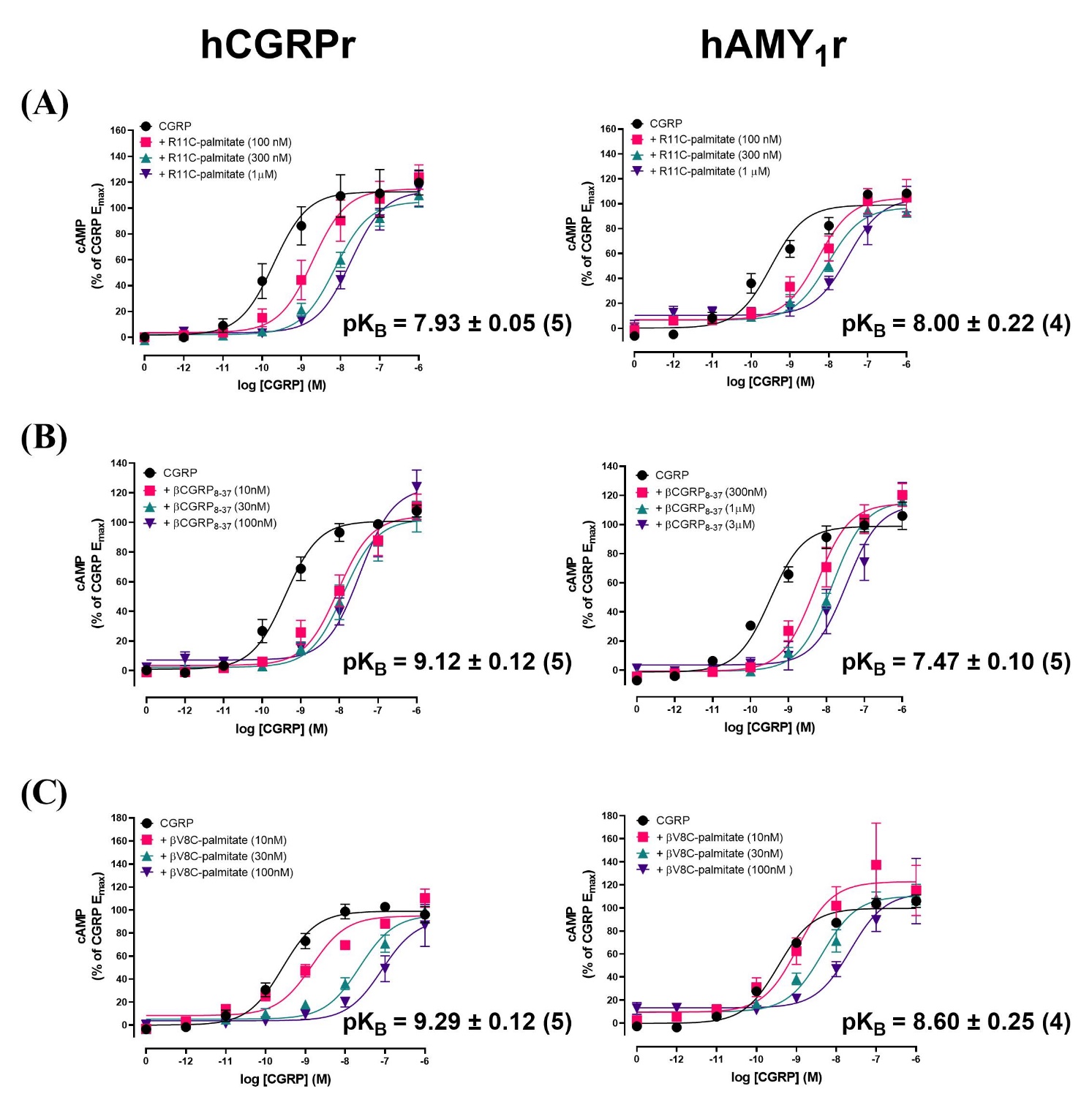


**Supplementary Fig. 7.** Antagonism of CGRP-stimulated cAMP production by lipidated α/βCGRP_8-37_ analogues at hCGRP or AMY_1_ receptors expressed in Cos-7 cells. Concentration-response curves were generated in the absence or presence of (A) αCGRP_8-37_ R11C-palmitate, (B) βCGRP_8-37_, and (C) βV8C-palmitate at multiple different concentrations. Data points are plotted as a percentage of maximal CGRP-stimulated cAMP production as mean ± SEM of 4-5 independent experiments. pK_B_ values are also presented as mean ± SEM (n).


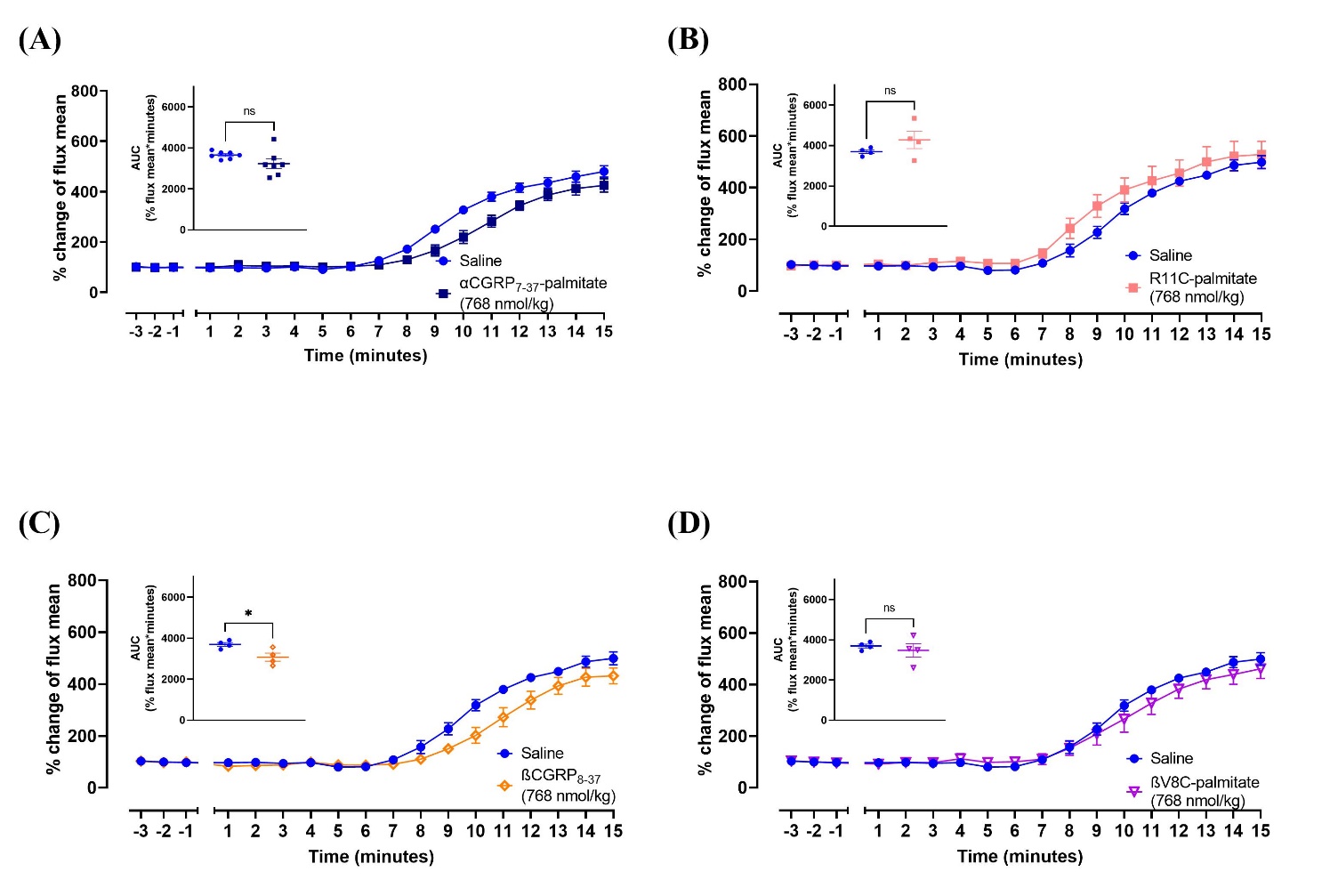


**Supplementary Figure 8.** Antagonist effects of (A) αCGRP_7-37_-palmitate, (B) R11C-palmitate, (C) βCGRP_8-37_, and (D) βV8C-palmitate on capsaicin-induced dermal vasodilation in male mice. Inset graphs show corresponding AUC mean values over the 15 minute measurement timeframe following capsaicin application to the ear. Each treatment group comprises 4-7 independent repeats with matched saline groups. Comparisons to saline were analyzed by unpaired Student’s t test.

## Supplementary Tables

**Supplementary Table 1.** pEC_50_ value of hCGRP and pA_2_ values of CGRP peptide analogues at h CGRP and AMY_1_ receptors expressed in Cos-7 cells. Values are mean ± SEM of three or (n) independent experiments. Comparisons of αCGRP pEC_50_ was analyzed by unpaired Student’s t-test. Comparisons of pA_2_ values for αCGRP_8-37_, αCGRP_7-37_, and cysteine-substituted peptide analogues were analyzed by one-way ANOVA followed by Dunnett’s multiple comparison test against αCGRP_8-37_. * p<0.05, ** p<0.01, *** p<0.001 significance.

|  | αCGRP | αCGRP_8-37_ | αCGRP_7-37_ | V8C αCGRP_8-37_ | K24C αCGRP_8-37_ | K35C αCGRP_8-37_ |
| --- | --- | --- | --- | --- | --- | --- |
| CGRPr | 9.80 ± 0.12 (6) | 8.63 ± 0.07 | 7.98 ± 0.09* | 7.23 ± 0.07*** | 8.25 ± 0.11 | 8.85 ± 0.31 |
| AMY_1_r | 9.39 ± 0.12* (6) | 7.08 ± 0.15 | 7.10 ± 0.17 | 6.41 ± 0.15* | 7.06 ± 0.15 | 7.04 ± 0.18 |

## Supplementary Chemistry for β-CGRP_8-37_, β-CGRP_8-37_ V8C(S-Pam) and α-CGRP_8-37_ R11C(S-Pam).

### General Procedure

All reagents were purchased as reagent grade and used without further purification. *N*,*N*-Diisopropylethylamine (DIPEA), piperidine, *N*,*N*′-diisopropylcarbodiimide (DIC),
1,2-ethanedithiol (EDT), triisopropylsilane (TIPS) and 4-methylmorpholine (NMM) were purchased from Sigma-Aldrich (St. Louis, Missouri). *O*-(7-Azabenzotriazol-1-yl)-*N*,*N*,*N*′,*N*′-tetramethyluronium hexafluorophosphate (HATU), Fmoc-Ala-OH, Fmoc-Arg(Pbf)-OH (Pbf = 2,2,4,6,7-pentamethyIdlhydrobenzofuran-5-sulfonyl), Fmoc-Asn(Trt)-OH (Trt = triphenylmethane), Fmoc-Gly-OH, Fmoc-His(Trt)-OH, Fmoc-Leu-OH, Fmoc-Lys(Boc)-OH (Boc = *tert*-butoxycarbonyl), Fmoc-Met-OH, Fmoc-Phe-OH, Fmoc-Pro-OH, Fmoc-Ser(*t*Bu)-OH (*t*Bu = *tert*-butyl), Fmoc-Thr(*t*Bu)-OH, Fmoc-Val-OH and 4-[(2,4-Dimethoxyphenyl)(Fmoc-amino)methyl]phenoxyacetic acid (Rink amide linker) were purchased from CS Bio (Shanghai, China). 6-Chloro-1-hydroxybenzotriazole (6-Cl-HOBt) and Fmoc-Leu-Ser(Psi(Me,Me)pro)-OH were purchased from Apptec (Louisville, Kentucky). Aminomethyl polystyrene resin was purchased from Rapp Polymere (Tübingen, Germany). Microwave reactions were carried out on a Biotage^®^ Initiator+ Alstra^TM^ (Uppsala, Sweden) automated peptide synthesizer. Semi-preparative/analytical RP-HPLC was performed on a Thermo Scientific (Waltham, MA) Dionex Ultimate 3000 HPLC equipped with a four channel UV detector at 210, 225, 254 and 280 nm using either an analytical column (Waters (Milford, MA) XTerra^®^ MS C18 (5 μm; 4.6 × 150 mm) at a flow rate of 1 mL min^-1^ or a Phenomenex^®^ semi-preparative column (Torrance, CA), Gemini C18, (5 μm; 10 × 250 mm) at a flow rate of 4 mL min^-1^. A suitably adjusted gradient of 5% B to 95% B was used, where solvent A was 0.1% TFA in H_2_O and B was 0.1% TFA in acetonitrile. LC-MS spectra were acquired using Agilent Technologies (Santa Clara, CA) 1260 Infinity LC equipped with an Agilent Technologies 6120 Quadrupole mass spectrometer. An analytical column (Agilent ZORBAX 300SB-C3, 3.5 μm; 3.0 × 150 mm) was used at a flow rate of 0.3 mL min^-1^ using a linear gradient of 5% B to 95% B over 30 min, where solvent A was 0.1% formic acid in H_2_O and B was 0.1% formic acid in acetonitrile.

### General Methods

**Method 1: General procedure for attachment of Fmoc Rink amide to the resin:**

To aminomethyl polystyrene resin (80 mg, 0.1 mmol, loading: 1.26 mmol/g) pre-swollen in CH_2_Cl_2_ (5 mL, 20 min), was added 4-[(2,4-dimethoxyphenyl)(Fmoc-amino)methyl]phenoxyacetic acid (220.2 mg, 4 equiv., 0.4 mmol) and 6-Cl-HOBt (70 mg, 3.5 equiv., 0.35 mmol) dissolved in DMF (1.5 mL) followed by addition of DIC (62 µL, 4 equiv., 0.4 mmol). The reaction mixture was gently agitated at room temperature for 24 h. The resin was filtered and washed with DMF (3 × 3 mL) after which a negative Kaiser test confirmed successful coupling**.^1^**

**Method 2: General procedure for removal of *N*^α^-Fmoc-protecting group:**

Peptidyl resin was treated with a solution of 20 vol % piperidine in DMF (*v*/*v*, 4 mL) and the mixture was agitated on the Biotage^®^ Initiator Alstra for 2 × 5 min at room temperature. The resin was filtered and washed with DMF (3 × 3 mL).

**Method 3:** **General coupling procedure for Fmoc-Ala-OH, Fmoc-Asn(Trt)-OH, Fmoc-Gly-OH, Fmoc-Leu-OH, Fmoc-Lys(Boc)-OH, Fmoc-Met-OH, Fmoc-Phe-OH, Fmoc-Pro-OH, Fmoc-Ser(*t*Bu)-OH, Fmoc-Thr(*t*Bu)-OH, Fmoc-Val-OH:**

Couplings were performed using the Biotage^®^ Initiator Alstra with the appropriate Fmoc-protected amino acid (0.2 M, DMF,
5 equiv.), HATU (0.5 M, DMF, 4.75 equiv.) and NMM in DMF (2 M, 8 equiv.) using a single coupling cycle at 75 °C,
110 W for 5 min. The resin was filtered and washed with DMF (3 × 3 mL).

**Method 4: General coupling procedure for Fmoc-Arg(Pbf)-OH:**

Double coupling cycles of Fmoc-Arg(Pbf)-OH were carried out with the Biotage^®^ Initiator Alstra using Fmoc-Arg(Pbf)-OH (0.2 M, DMF, 5 equiv.), HATU (0.5 M, DMF, 4.75 equiv.) and NMM in DMF (2 M, 8 equiv.) with first coupling at room temperature for 25 min, followed by a second coupling cycle using fresh reagents at 72 °C, 110 W for 5 min. The resin was filtered and washed with DMF (3 × 3 mL).

**Method 5: General coupling procedure for Fmoc-His(Trt)-OH:**

Double coupling cycles of Fmoc-His(Trt)-OH were carried out with the Biotage^®^ Initiator Alstra using Fmoc protected amino acid (0.2 M, DMF, 5 equiv.), HATU (0.5 M, DMF, 4.75 equiv.) and NMM in DMF (2 M,
8 equiv.) with first coupling at room temperature for 15 min, followed by a second coupling using fresh reagents at 43 °C, 110 W for 10 min. The resin was filtered and washed with DMF (3 × 3 mL).

**Method 6: Coupling of Fmoc-dipeptide(Psi(Me,Me)pro)-OH:**

Deprotected resin was treated with the appropriate Fmoc-dipeptide(Psi(Me,Me)pro)-OH (5 equiv., 0.5 mmol) and HATU (171.1 mg, 4.5 equiv., 0.45 mmol) in 1.5 mL DMF followed by addition of NMM (88 µL,
8 equiv., 0.8 mmol) and agitated for 30 min, before being filtered, washed with DMF (3 × 3 mL) and the procedure repeated again with fresh reagents. The resin was filtered and washed with DMF (3 × 3 mL).

**Method 7: General procedure for the capping of free amino groups:**

Fmoc-protected peptidyl resin was treated with 5 M Ac_2_O in DMF (0.47 mL, 2.5 equiv.) and NMM in DMF (2 M, 8 equiv.) using the Biotage^®^ Initiator Alstra at room temperature for 10 min. The resin was filtered and washed with DMF (3 × 3 mL).

**Method 8: General procedure for coupling of Fmoc-protected palmitoylated cysteine building block:^2^**

Deprotected resin was treated with Fmoc-Cys(*S*-Pam)-OH (157 mg, 2.5 equiv., 0.25 mmol) and HATU (72.2 mg, 1.9 equiv., 0.19 mmol) in 1.5 mL DMF followed by addition of *sym*-collidine (106 µL,
8 equiv., 0.8 mmol) and agitated for 40 min at room temperature, before being filtered, washed with DMF (3 × 3 mL) and the procedure repeated again with fresh reagents. The resin was filtered and washed with DMF (3 × 3 mL).

**Method 9: General procedure for TFA-mediated resin cleavage and global deprotection:**

Peptidyl resin was treated with a mixture of TFA/H_2_O/TIPS/EDT (94:2.5:2.5:1, *v/v/v/v*, 10 mL) for 120 min. The filtrate was partially concentrated under a gentle stream of N_2_, then cold diethyl ether was then added to form a precipitate. The mixture was centrifuged, and the solution was carefully decanted off and discarded, before dissolving the solid pellet in H_2_O:acetonitrile (1:1, *v*/*v,* 25 mL) containing 0.1% TFA and lyophilized.

### Synthesis of β-CGRP_8-37_ using Fmoc-SPPS.

**Scheme SC1. Synthesis of β-CGRP_8-37_.**


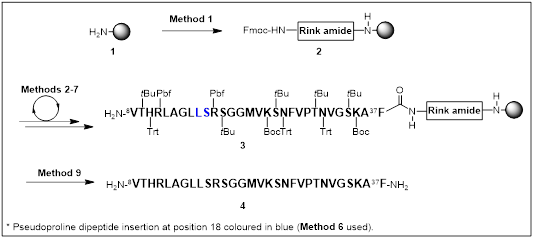


Synthesis of β-CGRP_8-37_ (**4**).

Fmoc-Rink amide was attached to aminomethyl polystyrene resin **1** using **Method 1** followed by Fmoc-removal using **Method 2**. Direct attachment of Fmoc-Phe-OH at position 37 to resin bound Fmoc-removed Rink amide **2** was achieved using **Method 3**. **Method 2** was used for all subsequent *N*^α^-Fmoc removals up to position ^8^Val. Linear elongation of the peptide chain was achieved by coupling appropriate Fmoc-amino acids up to ^18^Arg indicated in **Scheme SC1** using **Method 3**. All Fmoc-Arg(Pbf)-OH residues are coupled using **Method 4**. All Fmoc-His(Trt)-OH residues are coupled using **Method 5**. Capping of free amino groups was conducted throughout the synthesis using **Method 7**. Coupling of Fmoc-Leu-Ser(Psi(Me,Me)pro)-OH dipeptide at position 18 was achieved using **Method 6**. Linear synthesis of the peptide was continued up to Val at position 8 using appropriate **Methods** (**2**-**5**, **7**). Peptide **3** was then liberated from resin using **Method 9** affording **4**. Crude **4** was purified batchwise by semi-preparative RP-HPLC on a Phenomenex^®^ Gemini C18 column (10 × 250 mm, 5 μm) using a linear gradient of 5% to 95% over 90 min (*ca.* 1% B/min) with a flow rate of 4 mL/min. Fractions were collected at 0.2 min intervals and analyzed by ESI-MS and RP-HPLC. Fractions identified with correct *m*/*z* were combined and lyophilized to afford the *title compound* **4** as a white amorphous solid (5.1 mg, 1.6% yield based on 0.1 mmol loading, *t*_R_ = 25.2 min, *>* 99% purity); (**Figure SC1** and **SC2**).


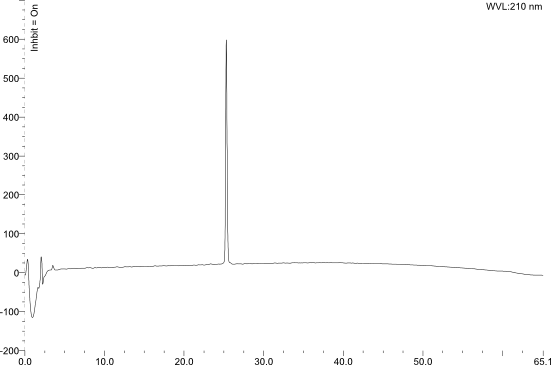


**Figure SC1**: Analytical RP-HPLC chromatogram of purified peptide **4**, *t*_R_ = 25.2 min. Chromatographic separations were performed on a Thermo Scientific Dionex Ultimate 3000 HPLC using a XTerra^®^ MS C-18 column (5 μm; 4.6 × 150 mm) and a linear gradient of 5-65% B in 65 min at room temperature, *ca*. 1% B per min at a flow rate of 1.0 mL/min. Buffer A: H_2_O containing 0.1% TFA (*v/v*); Buffer B: acetonitrile containing 0.1% TFA (*v/v*).


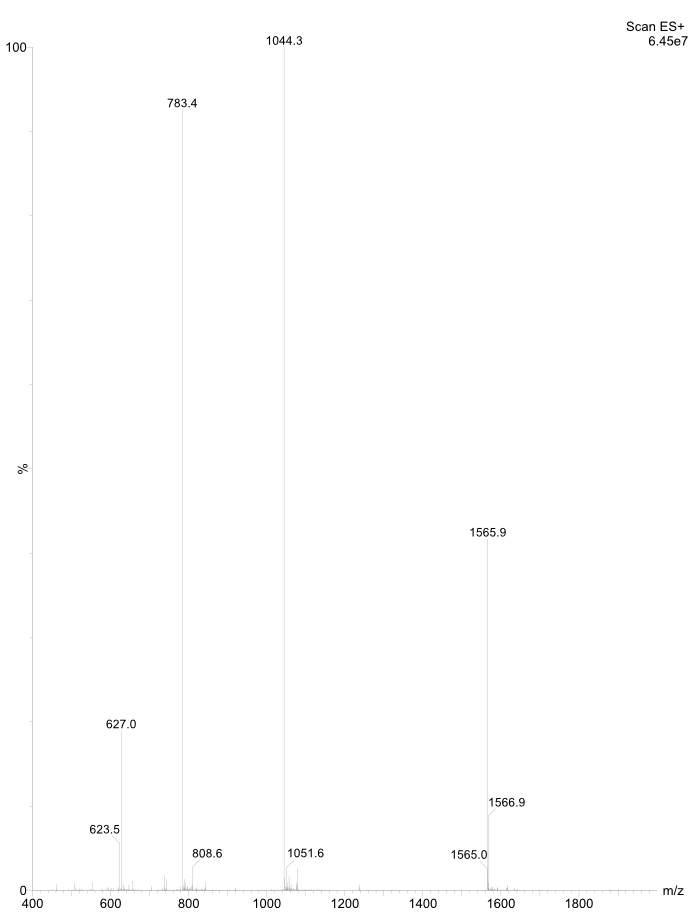


**Figure SC2:** FI-MS profile of purified peptide **4**; ion polarity positive operating at a nominal accelerating voltage of 20 eV. ESI-MS (*m/z* [M+2H]^2+^ calcd: 1566.3; found: 1565.9; [M+3H]^3+^ calcd: 1044.5; found: 1044.3; [M+4H]^4+^ calcd: 783.6; found: 783.4; [M+5H]^5+^ calcd: 627.1; found: 627.0. Mass deconvolution calculated at 3129.83 Da with standard deviation of 0.17; theoretical mass calculated at 3130.65 Da.

### Synthesis of β-CGRP_8-37_ V8C(S-Pam) using Fmoc-SPPS.

**Scheme SC2. Synthesis of β-CGRP_8-37_ V8C(S-Pam).**


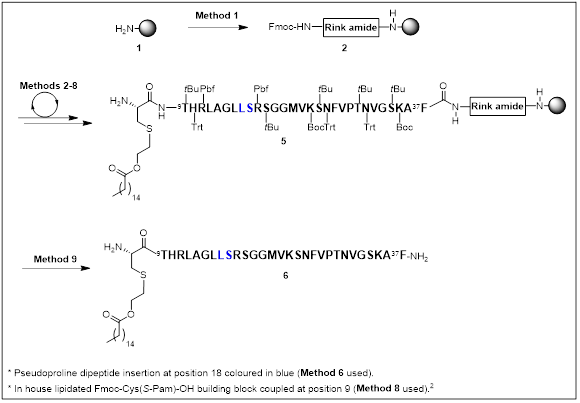


Synthesis of β-CGRP_8-37_ V8C(S-Pam) (**6**).

Fmoc-Rink amide was attached to aminomethyl polystyrene resin **1** using **Method 1** followed by Fmoc-removal using **Method 2**. Direct attachment of Fmoc-Phe-OH at position 37 to resin bound Fmoc-removed Rink amide **2** was achieved using **Method 3**. **Method 2** was used for all subsequent *N*^α^-Fmoc removals up to position ^8^Cys. Linear elongation of the peptide chain was achieved by coupling appropriate Fmoc-amino acids up to ^18^Arg indicated in **Scheme SC2** using **Method 3**. All Fmoc-Arg(Pbf)-OH residues are coupled using **Method 4**. All Fmoc-His(Trt)-OH residues are coupled using **Method 5**. Capping of free amino groups was conducted throughout the synthesis using **Method 7**. Coupling of Fmoc-Leu-Ser(Psi(Me,Me)pro)-OH dipeptide at position 18 was achieved using **Method 6**. Linear synthesis of the peptide was continued up to ^8^Cys using appropriate **Methods** (**2**-**5**, **7-8**). Peptide **5** was then liberated from resin using **Method 9** affording **6**. Crude **6** was purified batchwise by semi-preparative RP-HPLC on a Phenomenex^®^ Gemini C18 column (10 × 250 mm, 5 μm) using a linear gradient of 5% to 95% over 90 min (*ca.* 1% B/min) with a flow rate of 4 mL/min. Fractions were collected at 0.2 min intervals and analyzed by ESI-MS and RP-HPLC. Fractions identified with correct *m*/*z* were combined and lyophilized to afford the *title compound* **6** as a white amorphous solid (5.15 mg, 1.5% yield based on 0.1 mmol scale, *t*_R_ = 44.3 min, *>* 99% purity); (**Figure SC3** and **SC4**).


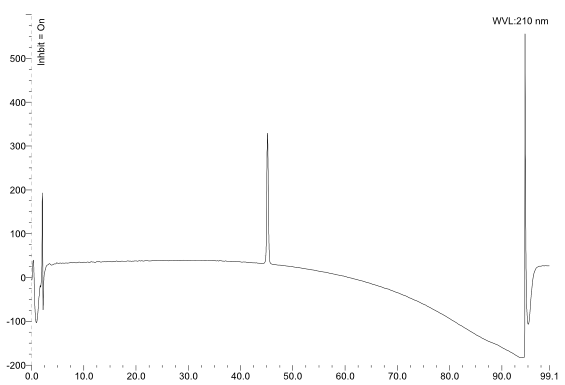


**Figure SC3**: Analytical RP-HPLC chromatogram of purified peptide **6**, *t*_R_ = 44.3 min. Chromatographic separations were performed on a Thermo Scientific Dionex Ultimate 3000 HPLC using a XTerra^®^ MS C-18 column (5 μm; 4.6 × 150 mm) and a linear gradient of 5-95% B in 90 min at room temperature, *ca*. 1% B per min at a flow rate of 1.0 mL/min. Buffer A: H_2_O containing 0.1% TFA (*v/v*); Buffer B: acetonitrile containing 0.1% TFA (*v/v*).


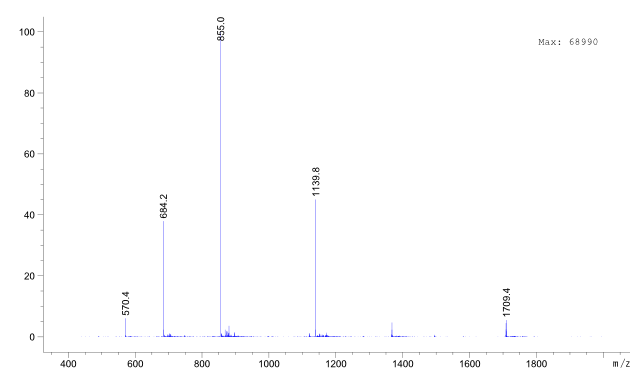


**Figure SC4**: FI-MS profile of purified peptide **6**; ion polarity positive operating at a nominal accelerating voltage of 75 eV. ESI-MS (m/z [M+2H]^2+^ calcd: 1709.5; found: 1709.4; [M+3H]^3+^ calcd: 1140.0; found: 1139.8; [M+4H]^4+^ calcd: 855.3; found: 855.0; [M+5H]^5+^ calcd: 684.4; found: 684.2; [M+6H]^6+^ calcd: 570.5; found: 570.4. Mass deconvolution calculated at 3416.32 Da with standard deviation of 0.33; theoretical mass calculated at 3417.14 Da.

### Synthesis of α-CGRP_8-37_ R11C(S-Pam) using Fmoc-SPPS

**Scheme SC3. Synthesis of α-CGRP_8-37_ R11C(S-Pam)**


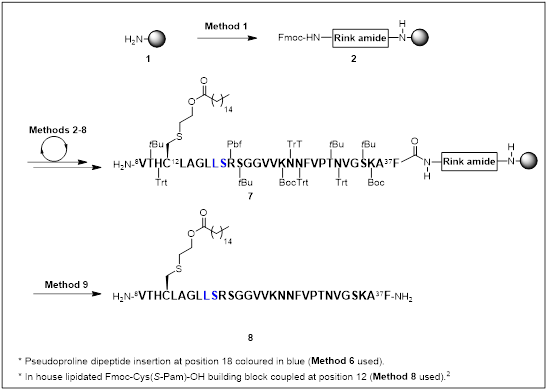


Synthesis of α-CGRP_8-37_ R11C(S-Pam) (**8**).

Fmoc-Rink amide was attached to aminomethyl polystyrene resin **1** using **Method 1** followed by Fmoc-removal using **Method 2**. Direct attachment of Fmoc-Phe-OH at position 37 to resin bound Fmoc-removed Rink amide **2** was achieved using **Method 3**. **Method 2** was used for all subsequent *N*^α^-Fmoc removals up to position ^8^Val. Linear elongation of the peptide chain was achieved by coupling appropriate Fmoc-amino acids up to ^18^Arg indicated in **Scheme SC3** using **Method 3**. All Fmoc-Arg(Pbf)-OH residues are coupled using **Method 4**. All Fmoc-His(Trt)-OH residues are coupled using **Method 5**. Capping of free amino groups was conducted throughout the synthesis using **Method 7**. Coupling of Fmoc-Leu-Ser(Psi(Me,Me)pro)-OH dipeptide at position 18 was achieved using **Method 6**. Linear synthesis of the peptide was continued up to ^8^Val using appropriate **Methods** (**2**-**5**, **7-8**). Peptide **7** was then liberated from resin using **Method 9** affording **8**. Crude **8** was purified batchwise by semi-preparative RP-HPLC on a Phenomenex^®^ Gemini C18 column (10 × 250 mm, 5 μm) using a linear gradient of 5% to 95% over 90 min (*ca.* 1% B/min) with a flow rate of 4 mL/min. Fractions were collected at 0.2 min intervals and analyzed by ESI-MS and RP-HPLC. Fractions identified with correct *m*/*z* were combined and lyophilized to afford the *title compound* **8** as a white amorphous solid (5.07 mg, 1.5% yield based on 0.1 mmol scale, *t*_R_ = 48.1 min, *>* 99% purity); (**Figure SC5** and **SC6**).


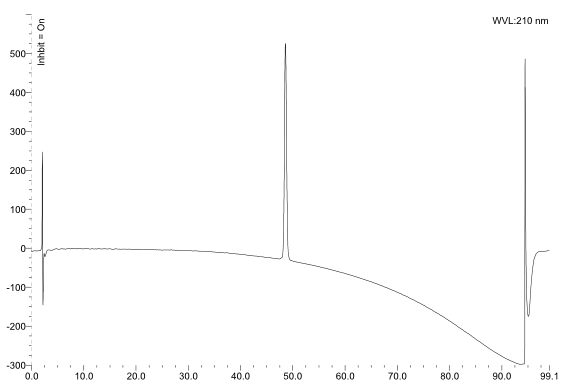


**Figure SC5**: Analytical RP-HPLC chromatogram of purified peptide **8**, *t*_R_ = 48.1 min. Chromatographic separations were performed on a Thermo Scientific Dionex Ultimate 3000 HPLC using a XTerra^®^ MS C-18 column (5 μm; 4.6 × 150 mm) and a linear gradient of 5-95% B in 90 min at room temperature, *ca*. 1% B per min at a flow rate of 1.0 mL/min. Buffer A: H_2_O containing 0.1% TFA (*v/v*); Buffer B: acetonitrile containing 0.1% TFA (*v/v*).


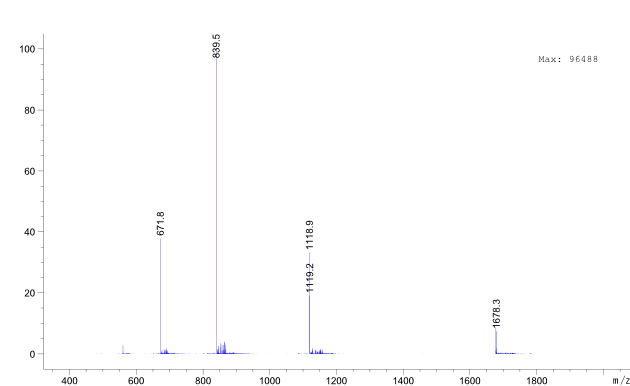


**Figure SC6**: FI-MS profile of purified peptide **8**; ion polarity positive operating at a nominal accelerating voltage of 75 eV. ESI-MS (m/z [M+2H]^2+^ calcd: 1678.5; found: 1678.3; [M+3H]^3+^ calcd: 1119.3; found: 1118.9; [M+4H]^4+^ calcd: 839.7; found: 839.5; [M+5H]^5+^ calcd: 672.0; found: 671.8. Mass deconvolution calculated at 3354.08 Da with standard deviation of 0.38; theoretical mass calculated at 3355.051 Da.

## References

(1) Kaiser, E.; Colescott, R. L.; Bossinger, C. D.; Cook, P. I. *Anal. Biochem*. **1970**, *34(2)*, 595-598.

(2) Lu, B. L.; Loomes, K. M.; Hay, D. L.; Harris, P. W. R.; Brimble, M. A. *J. Labelled Compd. Radiopharm.* **2020**, *63*, 325-332.
